# Comparative methylome analysis in solid tumors reveals aberrant methylation at chromosome 6p in nasopharyngeal carcinoma

**DOI:** 10.1002/cam4.451

**Published:** 2015-04-29

**Authors:** Wei Dai, Arthur Kwok Leung Cheung, Josephine Mun Yee Ko, Yue Cheng, Hong Zheng, Roger Kai Cheong Ngan, Wai Tong Ng, Anne Wing Mui Lee, Chun Chung Yau, Victor Ho Fu Lee, Maria Li Lung

**Affiliations:** 1 Department of Clinical Oncology, University of Hong KongHong Kong (SAR), China; 2 Center for Nasopharyngeal Carcinoma Research, University of Hong KongHong Kong (SAR), China; 3 Department of Clinical Oncology, Queen Elizabeth HospitalHong Kong (SAR), China; 4 Department of Clinical Oncology, Pamela Youde Nethersole Eastern HospitalHong Kong (SAR), China; 5 Department of Clinical Oncology, University of Hong Kong-Shenzhen HospitalShenzhen, China; 6 Department of Oncology, Princess Margaret HospitalHong Kong (SAR), China

**Keywords:** Chromosome 6p, EBV, Illumina HumanMethylation450, methylome, nasopharyngeal carcinoma

## Abstract

Altered patterns of DNA methylation are key features of cancer. Nasopharyngeal carcinoma (NPC) has the highest incidence in Southern China. Aberrant methylation at the promoter region of tumor suppressors is frequently reported in NPC; however, genome-wide methylation changes have not been comprehensively investigated. Therefore, we systematically analyzed methylome data in 25 primary NPC tumors and nontumor counterparts using a high-throughput approach with the Illumina HumanMethylation450 BeadChip. Comparatively, we examined the methylome data of 11 types of solid tumors collected by The Cancer Genome Atlas (TCGA). In NPC, the hypermethylation pattern was more dominant than hypomethylation and the majority of *de novo* methylated loci were within or close to CpG islands in tumors. The comparative methylome analysis reveals hypermethylation at chromosome 6p21.3 frequently occurred in NPC (false discovery rate; FDR=1.33 × 10^−9^), but was less obvious in other types of solid tumors except for prostate and Epstein–Barr virus (EBV)-positive gastric cancer (FDR<10^−3^). Bisulfite pyrosequencing results further confirmed the aberrant methylation at 6p in an additional patient cohort. Evident enrichment of the repressive mark H3K27me3 and active mark H3K4me3 derived from human embryonic stem cells were found at these regions, indicating both DNA methylation and histone modification function together, leading to epigenetic deregulation in NPC. Our study highlights the importance of epigenetic deregulation in NPC. Polycomb Complex 2 (PRC2), responsible for H3K27 trimethylation, is a promising therapeutic target. A key genomic region on 6p with aberrant methylation was identified. This region contains several important genes having potential use as biomarkers for NPC detection.

## Introduction

It is well-known that altered patterns of DNA methylation are key features of cancer. In particular, the cancer epigenome is characterized by global DNA hypomethylation and CpG island (CGI) promoter hypermethylation. Additionally, previous studies suggested that the methylation changes occurred at the CGI shores, regions close to CGI with comparatively lower CpG contents, and were important in cancers 1,2. It is known that the methylation alterations participate in the earliest stage of tumorigenesis 3,4. Therefore, systematic investigation of methylation changes in multiple types of cancers is necessary to further understand the role of altered methylation patterns in cancer initiation and development. Such comparative study can identify the commonly deregulated epigenetic machinery and discover the specific epigenetic mechanisms contributing to certain types of cancer. The Cancer Genome Atlas (TCGA) networks have carried out methylome studies in multiple types of cancers, and thus, provide a valuable data source for integrative methylation analysis across different types of cancers.

Nasopharyngeal carcinoma (NPC) is an Epstein–Barr virus (EBV)-associated epithelial malignancy that is the most prevalent in southern Chinese, but is relatively rare among Caucasians 5. In Hong Kong, the incidence of NPC is as high as 20–30 per 100,000 in men and 10–20 per 100,000 in women 6. Since EBV infection is ubiquitous in B lymphocytes, but is rare in epithelial cells 7, it is generally believed that other environmental and genetic factors are important determinants for NPC risk. Genetic variants at human leukocyte antigen (HLA) genes, located at the major histocompatibility complex (MHC) region on chromosome 6p21.3, showed the strongest association with NPC 8,9, indicating the importance of HLA region in NPC, although it remains unclear whether these genetic variants are disease causal or not.

Aberrant methylation is the most frequent event for gene silencing in NPC. For example, we and others have reported frequent promoter hypermethylation at *RASSF1*, *CDKN2A*, *DAPK*, *MIPOL1*, *WIF1*, *UCHL1*, *THY1*, and *PCDH10* in NPC 10–16. Recent reviews include more genes silenced by promoter hypermethylation in NPC 17,18. Furthermore, we have recently shown that aberrant methylation at the promoter region of four tumor suppressors provide a useful biomarker panel to detect NPC using noninvasive plasma 12.

In this study, we systematically examined the methylome data in NPC and matched nontumor adjacent tissues. Comparison of methylome data across solid tumors collected in TCGA studies, we found the *de novo* methylated loci in cancers often had H3K4me3 and H3K27me3 marks derived from human embryonic stem (ES) cells, suggesting a common epigenetic deregulation involves both DNA methylation and histone modification in NPC and other cancers. Furthermore, we identified a key genomic region at chromosome 6p21.3 frequently hypermethylated in NPC. Chromosome 6p21.3 is within MHC region where HLA resides. Several genes associated with *de novo* methylation from this region have excellent prospects for clinical application.

## Materials and Methods

### Clinical samples

All specimens were collected by the Area of Excellence (AoE) Hong Kong NPC Tissue Bank from Queen Mary, Queen Elizabeth, and Pamela Youde Nethersole Eastern Hospitals. The fresh-frozen tumor biopsies were hematoxylin and eosin (H&E) stained and subsequently reviewed to determine the tumor content. DNA extraction was performed using AllPrep DNA/RNA Micro Kit (Qiagen, Valencia, CA). The quality and quantity of the DNA samples was measured by NanoDrop (Thermo Scientific, Waltham, MA) and Qubit (Life Technologies, New York). In total, 25 NPC tumors were included in the discovery set and 35 NPC tumors in the validation set. The clinical parameters of the NPC patients in the discovery and validation sets are listed in Table S1. The power analysis showed we achieved about 80% of the power to detect the genes aberrantly methylated in 40% of the patients in the discovery set assuming a significance level of 5 × 10^−5^ (Fig. S1). With 35 NPC patients in the validation cohort there was about 90% of the power to validate the candidate loci methylated in at least 40% of the patients.

### DNA methylation profiling using Infinium Human 450k BeadArrays and data analysis

About 500 ng genomic DNA was bisulfite converted using EZ-96 DNA Methylation™ Kit (Zymo Research, Irvine, CA) following the manufacturer’s instructions. Illumina Infinium Assay analysis was performed by the Centre for Genomic Sciences at the University of Hong Kong following Illumina’s protocol. The raw data including signal intensities for methylated (M) and unmethylated (U) sequences were imported into Illumina GenomeStudio Methylation Module (Illumina, San Diego, CA). The bisulfite conversion efficiency and overall experimental efficiency were evaluated by the internal controls in GenomeStudio. The background signals were subtracted using average signal intensities of negative controls, then the signal intensities were normalized across the samples and converted to *β* value *β* = *M*/(*M* + *U* + 100) in GenomeStudio following the manufacturer’s manual. Subsequently, *β* values were imported into the R environment, where the following problematic probes were removed from the analysis: (1) the cross-reactive probes mapping to multiple locations on the genome and (2) the probes with low signals (detection *P* value > 0.01) in over 90% of the samples. Given two types of probes designed on the 450 k array display differing *β* value distributions, adjustment for probe design bias was applied using a beta-mixture quantile normalization method (BMIQ) 19. The corrected *β* values were transformed to *M*-values *M* = log_2_ (*β*/(1 − *β*)). Linear Models for Microarray Data (LIMMA) analysis 20 was used to detect the CpG sites differentially methylated between tumor and normal tissues. The significance level of multiple testing was adjusted using false discovery rate (FDR). The CpG sites with FDR < 0.001 in LIMMA analysis were defined as the loci differentially methylated in tumors. In each pair, the CpG sites were identified as hyper- or hypo-methylated loci, if the differential methylation between tumor and normal tissue was greater than 0.2 (¦Δ*β*¦ > 0.2). The unmethylated loci in normal tissues (average *β*_normal_ < 0.3) and peripheral blood mononuclear cells (PBMCs) (average *β*_pbmc_ < 0.3), but becoming methylated in at least 40% of the tumor pairs (Δ*β* > 0.2), were identified as the *de novo* methylated loci. The methylome data for PBMCs were obtained from 100 healthy females collected by the Marmal-aid database 21.

### Meta-analysis of methylome data in eleven types of solid tumors

The normalized methylome data on HumanMethylation450 BeadArray from eleven types of solid tumors, including prostate, breast, pancreatic, kidney, thyroid, liver, rectal, colon, head and neck, gastric, and lung cancers, were obtained from Marmal-aid database 21. Only the samples collected in TCGA studies were used in the study. LIMMA analysis for group comparison was used to identify the differentially methylated loci in each type of cancer, except gastric cancer, since only two normal gastric samples were available for the analysis. The number of tumor and normal tissues from each type of cancer is shown in Table S2. The CpG sites with FDR < 0.001 in LIMMA analysis were defined as the loci differentially methylated in tumors. The loci unmethylated in normal tissues (average *β*_normal_ < 0.3) and PBMCs (average *β*_pbmc_ < 0.3), but methylated in at least 40% of the tumors (*β*_tumor_ > 0.2) were identified as the *de novo* methylated loci. All data analysis was performed using the high-performance computing facility (HPCF) provided by the Centre of Genomic Sciences at the University of Hong Kong.

### DAVID functional analysis of the aberrantly methylated loci

The annotation of the *de novo* methylated loci was obtained from Illumina BeadStudio. Only the loci within or close to CGI were included in the analysis. The functions of the genes relevant to those loci were analyzed using the DAVID bioinformatics resources 22. The list of genes represented as RefSeq ID on HumanMethylation450 BeadChip was used as the background list to estimate the fold of the enrichment.

### Enrichment analysis of histone modification marks

The H3K4me3 and H3K27me3 histone modification peaks, as well as EZH2 and SUZ12 targets derived from human ES cells, were downloaded from the UCSC genome browser 23. Fisher’s exact test was used to evaluate the enrichment of each mark in the aberrantly methylated loci in each type of solid tumors.

### Identification of region-specific differential methylation in solid tumors

We developed an algorithm to detect the aberrant methylation within the large genomic regions including multiple CpG sites significantly hypermethylated in tumors. The method is based on the assumption that adjacent CpG sites within 50 bp have a similar methylation level 24, therefore, the adjacent CpG sites with FDR < 0.05 within 50 bp were merged together as a region, then the FDR of a region was estimated as a multiplicative FDR calculated from FDRs of all the CpG sites within the region. Since the array-based method has design bias toward the regions with high density of the genes, we corrected the FDR of a region by considering the total number of CpG sites designed in the assay (Supplementary Methods). The analysis was performed for each type of tumor. To compare the location of the regions aberrantly methylated across tumor types, only the top 500 regions ranking on the significant level were selected for the analysis.

### Gene expression data analysis

The expression data set (GSE12452) was downloaded from GEO database including 10 normal NP tissues and 31 laser-captured and microdissected NPC tissues. The raw data were processed using R package affy and normalized using the RMA method 25.

### Bisulfite pyrosequencing

To validate the aberrantly methylated loci, we performed bisulfite pyrosequencing. About 150 ng DNA in the discovery and validation sets was bisulfite converted using EZ DNA Methylation-Lightning™ Kit (Zymo Research). The completeness of bisulfite conversion was evaluated using Calponin PCR 26. The bisulfite-converted DNA was amplified using biotin-labeled PCR primers. The custom-designed PyroMark assay IDs (Qiagen) and PCR conditions for the selected loci are listed in Table S3. Bisulfite-converted CpGenome universal methylated DNA and unmethylated DNA (Millipore, Billerica, MA) were used as methylated and unmethylated controls. Bisulfite pyrosequencing was performed by the Centre of Genomics Sciences in the University of Hong Kong using PSQ96MA (Biotage, Charlotte, NC) as previously described 27.

### Data access

The NPC methylome data are available in NCBI GEO database with accession ID GSE62336.

## Results

### Identification of aberrant methylation in solid tumors

**Figure 1:**
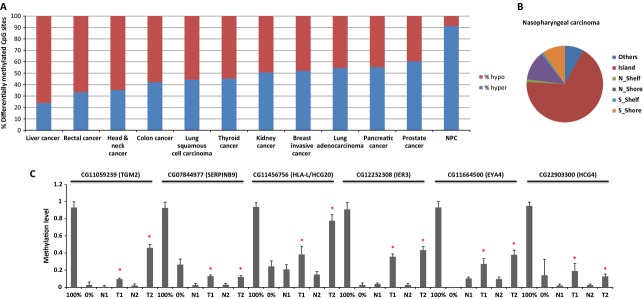
Aberrant methylation in NPC and other types of solid tumors. (A) Percentage of hypermethylated and hypomethylated CpG sites identified by LIMMA analysis with FDR < 0.001 in NPC and other types of solid tumors. hypo: hypomethylated CpG sites, hyper: hypermethylated CpG sites. (B) Categories of the genomic locations of *de novo* methylated CpG sites in NPC. Island: CpG island, N_Shelf: north shelf region of CGI, N_shore: north shore region of CGI, S_Shelf: south shelf region of CGI, N_Shelf: north shelf region of CGI. (C) Verification of the aberrant methylation of six selected loci identified in NPC by bisulfite pyrosequencing. 100% methylation control; 0% no methylation control; N: normal adjacent tissues, T: NPC tumor tissues. *Methylation level in tumor is significantly higher than matched nontumor tissue examined by Mann–Whitney *U*-test with *P* < 0.05. NPC, nasopharyngeal carcinoma; FDR, false discovery rate; CGI, CpG island.

Using the Illumina Infinium Assay on HumanMethylation450 BeadChip, we obtained methylation profiles of primary tumors and adjacent nontumor tissues from 25 NPC patients. The methylome data of prostate, breast, pancreatic, kidney, thyroid, liver, rectal, colon, lung, head and neck, and gastric cancers were generated in TCGA projects and obtained from Marmalaid database as detailed in Methods. LIMMA analysis was applied to identify the differentially methylated loci. We found the percentage of the hypermethylated loci out of total number of differentially methylated CpG sites with FDR < 0.001 in LIMMA analysis was variable across cancer types, ranging from 24% to 91% (Fig.1A). Hypermethylation was extremely frequent in NPC. Of 61825 CpG sites differentially methylated in NPC, 52967 (91%) of the CpG sites were hypermethylated, while only 5065 (9%) of CpG sites became significantly hypomethylated. In contrast, liver cancer had reduced methylation in 76% of CpG sites that lost methylation and only 24% of CpG sites had increased methylation levels in tumors.

Tumor tissues were often infiltrated with lymphocytes, therefore, in each cancer type we excluded the CpG sites methylated in PBMCs (*β *> 0.3). Meanwhile, the CpG sites methylated in normal adjacent tissues (*β *> 0.3) or methylated in less than 40% of the tumors were also removed from the analysis. In NPC, 5908 *de novo* methylated autosomal CpG sites corresponding to 2069 unique genes remained and 90.4% of these loci were within or close to CGI (CGI shore) (Fig.1B), which is much higher than the average level (72.3%) across 11 types of solid tumors (Fig. S2). Previously, we found the promoter regions of *RASSF1* and *WIF1* were methylated in over 60% of the NPC patients 12. Consistently, multiple CpG sites from promoter CGI of these two genes were identified as significant hits in the current study.

We selected six *de novo* methylated loci close to the genes of interest and examined the methylation level in the tumors from the discovery set by an independent method, bisulfite pyrosequencing. All six loci showed increased methylation in the tumors compared to the nontumor tissues, indicating a high accuracy for identifying aberrant methylation in our study (Figs.1C and S3).

### Significant enrichment of bivalent marks at *de novo* methylated loci in NPC and other solid tumors

To identify the molecular function of the genes relevant to the *de novo* methylated loci within CGIs or CGI shores in NPC, we performed the functional enrichment analysis using DAVID bioinformatics resources. The calcium signaling pathway, wnt signaling pathway, focal adhesion, phosphatidylinositol signaling system, regulation of actin cytoskeleton, mitogen-activated protein kinase (MAPK) signaling pathway, TGF-beta signaling pathway, gap junction, and hedgehog signaling pathway are often deregulated through aberrant methylation in NPC (adjusted *P* < 0.2) (Table S4). Notably, these aberrantly methylated genes frequently included HOX genes (FDR = 4.1 × 10^−26^) or other homeobox genes (FDR = 8.1 × 10^−32^). The homeobox genes often function as transcription factors; we observed a dramatic enrichment of transcription factor activity in the analysis (FDR = 4.4 × 10^−28^).

**Figure 2:**
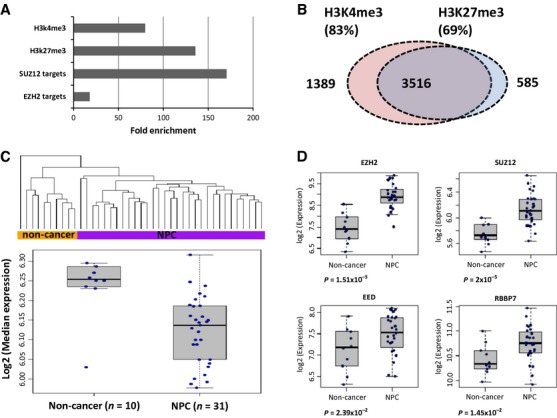
Enrichment of bivalent marks at the *de novo* methylated loci and overexpression of polycomb complex 2 genes in NPC. (A) The de novo methylated loci were more likely to be EZH2 and SUZ12 targets, and often had active H3K4 trimethylation marks and repressive H3K27 trimethylation marks derived from hESCs. (B) Significant overlap between the *de novo* methylated loci with H3K27me3 and H3K4me3. (C) Genes with bivalent marks and associated with *de novo* methylated loci were downregulated in NPC. Top: Unsupervised hierarchical clustering using expression data of the selected genes with bivalent marks and aberrantly methylated in NPC. The expression profiles of these genes can separate the majority of the normal controls from NPC tissues; Bottom: Median expression of bivalent genes in normal controls and NPC tissues. (D) Overexpression of *EZH2*, *SUZ12*, *EED,* and *RBBP7* in NPC. All the expression data were obtained from GEO data set GSE12452. NPC, nasopharyngeal carcinoma.

DNA methylation and histone modification are dependent on one another in regulating gene expression. In normal tissues, a number of genes have the active chromatin mark H3K4me3 and repressive chromatin mark H3K27me3, the so called bivalent marks. These genes are often strong targets of DNA methylation in cancers. HOX clusters often display both bivalent marks in pluripotent ES cells, thus, playing an important role in development and differentiation. Given a subset of HOX genes was found to be aberrantly methylated in NPC and there is crosstalk between DNA methylation and histone modifications, it is likely that the aberrantly methylated genes in NPC are often the targets of bivalent marks. Consistent with this hypothesis, there is noticeable enrichment of H3K4me3 and H3K27me3 marks derived from hESCs in the *de novo* methylated loci in NPC and there was obvious overlap between the loci with H3K4me3 and H3K27me3 marks (Fig.2A and B). Significant enrichment of bivalent marks at the aberrantly methylated loci is not only observed in NPC, but also noted in all other types of cancers examined in this study, indicating the common epigenetic deregulation involved in cancer development (Fig. S4). We examined the expression level of the genes associated with the aberrantly methylated loci with bivalent marks using the public expression data set from laser-captured microdissected NPC samples (GSE12452). Expression of these genes can separate the majority of the normal tissues from NPC tumor tissues using the unsupervised hierarchical clustering method, as shown in Figure2C. There was significant downregulation of this set of genes in NPC compared to normal adjacent tissues (LIMMA Mean-rank Gene Set Test one-sided *P* = 1.8 × 10^−29^), presumably through epigenetic regulation.

DNA methylation and H3K27me3 often function together in gene silencing 28. Polycomb complex 2 (PRC2) is mainly responsible for H3K27me3 29. The core human PRC2 complex comprises four components: EZH1/2, EED, SUZ12, and RBBP7/4 30. Consistent with enrichment of H3K27me3 marks in aberrant methylation, we also found the enrichment of PRC2 major players EZH2 and SUZ12 targets at these loci. Interestingly, *EZH2*, *EED*, *SUZ12*, and *RBBP7* were overexpressed at the mRNA level in NPC (Fig.2D). These *de novo* methylated loci may be the potential PRC2 targets in NPC.

### Frequent occurrence of aberrant methylation at 6p in NPC

**Figure 3:**
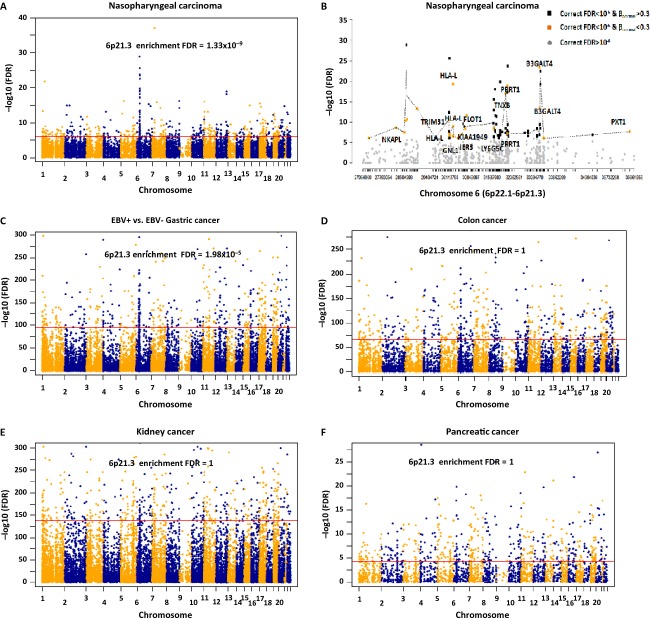
Aberrantly methylated regions identified in NPC and other types of solid tumors. (A) Aberrantly methylated regions across genome in NPC. Enrichment of the genes from 6p21.3 was identified by DAVID analysis. (B) Aberrantly methylated regions on 6p22.1-6p21.3 in NPC. Gray dots: the regions not reaching the significance level. Yellow dots: the regions reaching the significance level and which were unmethylated in normal adjacent tissues. The genes close to these regions are highlighted on the graph. Black dots: the regions reaching the significance level, but were methylated in normal adjacent tissues. (C) Aberrantly methylated regions in EBV-positive gastric cancer compared to EBV-negative gastric cancer. Enrichment of the genes from 6p21.3 was identified by DAVID analysis. (D–F) Aberrantly methylated regions in colon, kidney, and pancreatic cancers. No enrichment of the genes from 6p21.3 was observed. NPC, nasopharyngeal carcinoma; EBV, Epstein–Barr virus.

**Figure 4:**
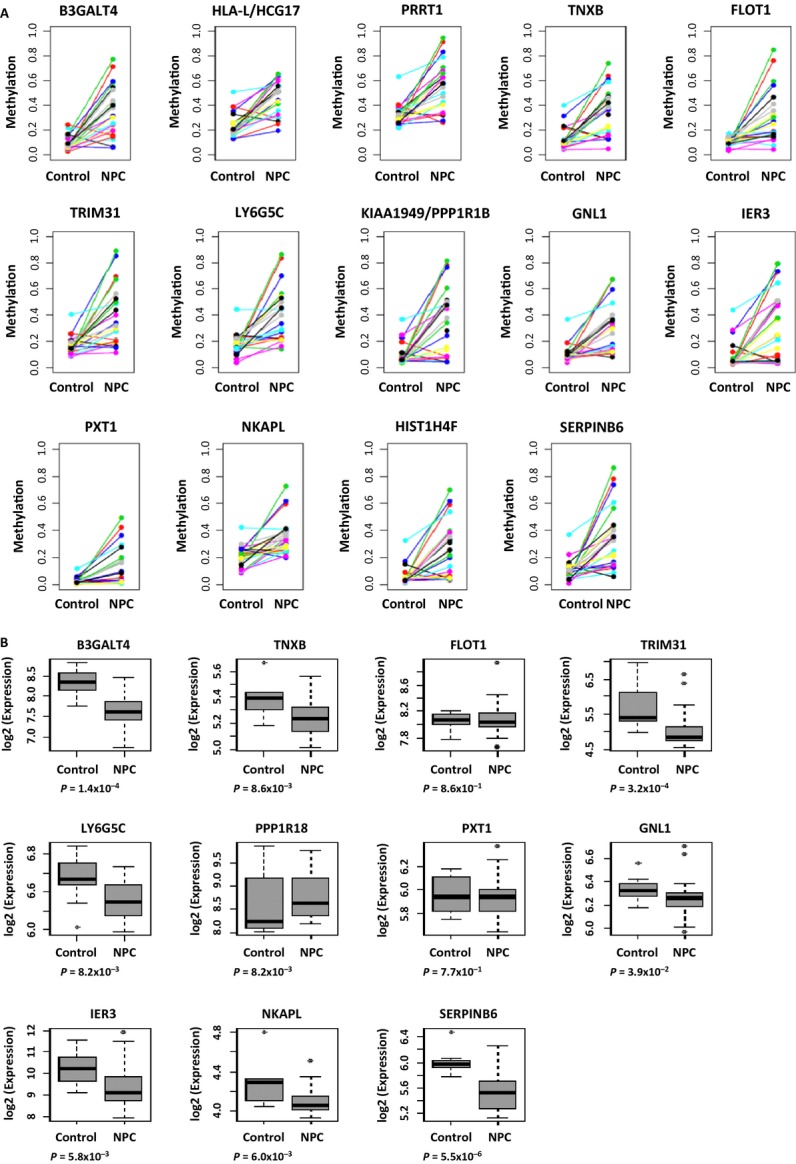
Methylation and expression of the genes from chromosome 6p in NPC. (A) Methylation level measured by Illumina Infinium Assay in normal tissues (control) and NPC tissues at the genes associated with *de novo* methylated regions from chromosome 6p. (B) We identified the genes aberrantly methylated in NPC from chromosome 6p and examined the expression level of these genes in normal tissues (control) and NPC tissues. There was downregulation of expression at genes *B3GALT4*, *TNXB, TRIM31, LY6G5C*, *GNL1*, *IER3*, *NKAPL,* and *SERPINB6* in NPC (Mann–Whitney *U*-test *P* < 0.05), presumably through epigenetic regulation. Genes *HLA-L/HCG20*, *PRRT1,* and *HIST1HF4* were not present on the Affymetrix Human U133Plus2 array, and thus, were excluded in this analysis. The *P* value was estimated by Mann–Whitney *U*-test. NPC, nasopharyngeal carcinoma.

Aberrant methylation often occurs at specific regions and there is a CGI methylator phenotype in cancers 31. To determine whether there is any specific genomic region with higher hypermethylation frequencies in tumors, we merged the individual adjacent CpG sites into small regions as described in the Methods. Figure3A shows the significant level of the regions with multiple hypermethylated loci across 22 chromosomes in NPC. Of the top 500 regions hypermethylated in NPC (FDR < 10^−6^), 76 regions (15.2%) localized to chromosome 6p (Fig.3AA and B). Table S5 lists 256 important regions selected from the top 500 regions, which were *de novo* methylated in NPC (average *β*_normal_ < 0.3 and average *β*_pbmc_ < 0.3). The genes with *de novo* methylation on 6p in NPC include *B3GATL4* (6p21.3), *HLA-L/HCG20* (6p21.3), *PRRT1* (6p21.32), *TNXB* (6p21.3), *FLOT1* (6p21.3), *TRIM31* (6p21.3), *LY6G5C* (6p21.33), *KIAA1949*/*PPP1R18* (6p21.3), *GNL1* (6p21.3)*, IER3* (6p21.3), *PXT1* (6p21.31), *NKAPL* (6p22.1), *HIST1H4F* (6p22.1), and *SERPINB6* (6p25) (Fig.4A). Microarray data (GEO accession ID: GSE12452) showed reduction in expression at *B3GALT4*, *TNXB*, *TRIM31*, *LY6G5C*, *GNL1*, *IER3*, *NKAPL*, and *SERPINB6* in NPC (*P* < 0.05, Fig.4B), presumably through epigenetic regulation. Again, these genes often contain H3K27me3 and H3K4me3 marks derived from hESCs (Table S5), indicating histone modification plays a role in gene silencing in this region as well.

The genes associated with the top 500 hypermethylated regions were frequently located at 6p21.3 in NPC (FDR = 1.33 × 10^−9^) (Fig.3A and B). The permutation test showed it was unlikely this observed peak occurred by chance or was due to any array design bias (Fig. S5). Similarly, the analysis revealed there was a dramatic increase in methylation level at 6p21.3 in EBV-positive gastric tumors (*n* = 19) compared to EBV-negative tumors (*n* = 151) (FDR = 1.98 × 10^−5^, Fig.3C). We did not observe the ethnic difference of Asian versus Caucasian populations in the EBV-positive and EBV-negative gastric cancer patients (EBV-positive *N* = 19, Asian 31.6%, Caucasian 68.4%; EBV-negative *N* = 159, Asian 20.5%, Caucasian 64.2%, chi-square test *P* = 0.68). The especially frequent aberrant methylation at this region may be relevant to EBV infection, but more studies are required to further investigate this interesting finding. Although in other types of solid tumors, we also observed aberrant methylation in a few small regions at 6p hypermethylated in tumors, however, only prostate cancer also had statistically significant enrichment of the genes hypermethylated at 6p21.3 (Figs.3D–F and S6).

**Figure 5:**
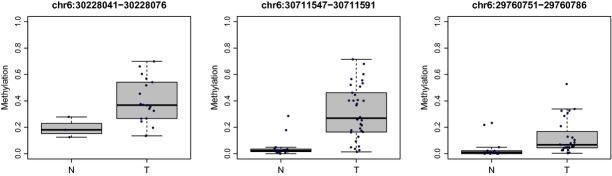
Bisulfite pyrosequencing of the selected loci from 6p21.3 in the validation set. Aberrant methylation at the selected loci from 6p21.3 was further examined by bisulfite pyrosequencing in an additional NPC patient cohort. The *P* value was estimated using the Mann–Whitney *U*-test. *P < 0.05; ***P < 0.001; ****P < 0.0001. NPC, nasopharyngeal carcinoma.

We further investigated the methylation level of three selected loci from 6p21.3 in an independent patient cohort using bisulfite pyrosequencing. Figure5 shows dramatic elevation of methylation at these *de novo* methylated loci in NPC tumors compared to the nontumor adjacent tissues.

## Discussion

In this study, we investigated genome-wide methylation changes in NPC tissues compared to their normal counterparts using a high-throughput approach, and performed meta-analysis of methylome data in other types of solid tumors generated in TCGA studies. Compared to other types of cancers, NPC had a higher hypermethylation frequency within CGI or close to CGI and very few CpG sites were significantly hypomethylated in NPC, indicating hypermethylation is the most frequent event in NPC. Our results showed hypomethylation was less frequent in NPC. However, the HumanMethylation450 BeadChip does not cover the repetitive elements and the regions with poor coverage of the genes. Therefore, methylation changes at these regions were not investigated due to the limitation of the array design.

Hypermethylation occurred across the NPC genome, but was more striking on chromosome 6p, especially at chromosome 6p21.3. The MHC region, which is highly polymorphic and has a high density of genes 32, is located in this region. Previous GWAS studies showed multiple loci (*HLA-A*, *HLA-F* and *GABBR1*) within 6p21.3 were associated with NPC 8,9. In this analysis, we excluded the probes targeting the sequences containing the common single-nucleotide polymorphisms (SNPs) or within 10 bp away from the common SNPs. So it is unlikely that the hypermethylated regions identified from this study were due to the polymorphic CpG sites. Aberrant methylation at the genes *B3GATL4* (6p21.3), *TNXB* (6p21.3), *TRIM31* (6p21.3), *LY6G5C* (6p21.33), *KIAA1949*/*PPP1R18* (6p21.3), and *GNL1* (6p21.3) identified in NPC was also hypermethylated in EBV-positive gastric cancers compared to EBV-negative gastric cancers (FDR < 1.6 × 10^−90^). Several previously reported findings imply that EBV infection functions as an epigenetic driver for tumorigenesis: (1) EBV-encoded latent membrane protein 1 (LMP1) could induce expression of DNA methyltransferases in NPC cells and in vitro EBV infection led to extensive DNA methylation 33,34; (2) EBV-positive gastric cancer showed a distinct high hypermethylation profile 34–36; (3) EBV infection of immortalized oral keratinocytes induced a hypermethylator phenotype 37; and (4) EBV could manipulate the polycomb group protein responsible for epigenetic repression of transcription 38. Further investigation in EBV-associated NPC is necessary to fully understand how EBV interacts with the host genome to affect host genome methylation, but is beyond the scope of the current study.

None of the genes associated with *de novo* methylation from 6p21.3 have been studied in NPC. For example, *B3GALT4* belongs to the beta-1,3-galactosyltransferase gene family. This gene family encodes type II membrane-bound glycoproteins. *B3GALT4* is located in the centromeric segment of the human MHC class II region. Although the associations between B3GALT4 and ovarian and uterine corpus cancer, as well as melanoma have been reported 39,40, the function of this gene is not fully understood in cancers. We found decreased expression of *B3GALT4* was associated with poor disease-free survival in both ovarian and lung cancers (ovarian cancer: HR = 0.78, 95% CI 0.67–0.89, *P* = 0.00043; lung cancer: HR = 0.73, 95% CI 0.58–0.92, *P* = 0.0075, Fig. S7), indicating the likelihood that this gene plays a role in cancer progression.

In our previous study, we have shown a methylation gene panel including *RASSF1*, *WIF1*, *RARB2*, and *DAPK1* has great potential to be used as a noninvasive and complementary test for NPC early detection in combination with the EBV DNA test 12. Although we increased the detection rate of early-stage NPC patients from 51.2% (EBV DNA test alone) to 64.6% (methylation panel and EBV DNA test), still more than 30% of early-stage NPC patients cannot be identified. In this study, we found methylation of a locus (chr6: 30711547–30711591) from 6p21.3 region occurred in 76.5% (13/17) of the early-stage patients from discovery and validation sets. Therefore, combination of our methylation gene panel and the novel aberrantly methylated loci identified in this current study, especially those loci located at 6p21.3, might further improve the detection rate for early-stage patients.

After integration of cancer methylome data and hESC ChipSeq data, we found significant overlap between *de novo* methylated loci and histone modification marks H3K4me3 and H3K27me3 across all types of cancers examined in this study. PRC2 places H3K27 trimethylation marks and is involved in various biological processes including differentiation, proliferation, and stem cell plasticity 29. The association between *de novo* methylation and bivalent markers derived from hESCs potentially links cancer development to the stem cell theory 31. Corresponding to methylome data, expression of *EZH2* and other PRC2 members increased in NPC and many other types of cancers, including breast, prostate, kidney, lung, bladder, and liver cancers 41–44, and are promising therapeutic targets in cancer therapy. Due to the limitation of material obtained from NPC biopsies, we did not directly examine H3K4me3 and H3K27me3 status in NPC tissues. It would be necessary to examine both marks systematically to establish the direct link between DNA methylation and histone modifications in NPC. Furthermore, small molecule inhibitors of EZH2 catalytic activity have been developed and tested in a variety of cancers. However, EZH2 inhibitors have not been tested in NPC. Further evaluation of the use of EZH2 inhibitors in NPC treatment is warranted.

Our study highlights the importance of epigenetic deregulation in NPC. An important genomic region on 6p with aberrant methylation was identified. This region contains several important genes having potential use as biomarkers for NPC detection.

## Supporting Information

**Figure S1.** Power estimation in the discovery set. **Figure S2.** Category of *de novo* methylated CpG sites in solid tumors. **Figure S3.** Bisulfite pyrosequencing of six *de novo* methylated loci identified by Illumina Infinium Assay in NPC. **Figure S4.** Enrichment of H3K27me3 and H3K4me3 at the *de novo* methylated loci. **Figure S5.** Permutation test in the region-specific analysis in NPC. **Figure S6.** Region-specific analysis of methylome data. **Figure S7.** Expression of B3GALT4 and disease-free survival in ovarian cancer (left) and lung cancer (right). **Table S1.** Clinical parameters of the NPC patients in discovery and validation sets. **Table S2.** Differential methylation in 10 types of solid tumors collected by TCGA. **Table S3.** Bisulfite pyrosequencing primers and PCR conditions. **Table S4.** KEGG pathways associated with *de novo* methylation in NPC. **Table S5.** De novo methylated regions in NPC.
