# Supplementary material for: Comparative methylome analysis in solid tumors reveals aberrant methylation at chromosome 6p in nasopharyngeal carcinoma
Source: Cancer Med. 2015 Apr 29;4(7):1079–90. doi: 10.1002/cam4.451 (PMC4529346; doi:10.1002/cam4.451)
Supplement: Supplementary file 1 [file cam40004-1079-sd1.pdf]

Supplementary Table S1: Clinical parameters of the NPC patients in discovery and validation sets

	Discovery set (N=25)	Validation set (N=35)
**Age (95% CI)**	51 (46,55)	53 (48,58)
**Gender**		
Female	5	6
Male	20	28
unknown	0	1
**Stage**		
I	3	4
II	2	10
III	13	11
IV	7	9
unknown	0	1

Supplementary Table S2: Differential methylation in 10 types of solid tumours collected by TCGA

Index	Type of Cancer	# Normal	# Tumour	# hyper	# hypo	% hyper	% hypo
1	Liver cancer	50	98	40763	129619	23.92%	76.08%
2	Rectal cancer	7	90	15240	30447	33.36%	66.64%
3	Colon cancer	38	258	51763	71013	42.16%	57.84%
4	Head & neck cancer	50	310	76763	141180	35.22%	65.78%
5	Lung squamous cell carcinoma	75	227	126015	157598	44.43%	55.57%
6	Thyroid cancer	56	200	24300	29447	45.21%	54.79%
7	Kidney cancer	183	200	135296	131387	50.73%	49.27%
8	Breast invasive cancer	98	200	112249	103039	52.14%	47.86%
9	Lung adenocarcinoma	75	340	146526	122285	54.51%	45.49%
10	Pancreatic cancer	7	49	10646	8580	55.37%	44.63%
11	Prostate cancer	45	176	85308	56043	60.35%	39.65%
	Total	684	2148				

Supplementary Table S3: Bisulphite pyrosequencing primers and PCR conditions

Probe ID	gene symbol	Assay name	Amplicon length	Biotin modification on	Location	Number of CpGs	PCR conditions
CG11059239	TGM2	Hs_CG11059239_01_PM	307	Reverse primer	chr20:36793478-36793785	8	95°C 6min,45 cycles (95°C 30 sec, 56°C 30 sec and 70°C 30 sec), 72°C 5 min
CG07844977	SERPINB9	Hs_CG07844977_01_PM	310	Reverse Primer	chr6:2903505-2903815	6	95°C 6min,45 cycles (95°C 30 sec, 54°C 30 sec and 70°C 30 sec), 72°C 5 min
CG11456756	HLA-L	Hs_CG11456756_03_PM	210	Reverse primer	chr6:30227917-30228127	6	95°C 6min,45 cycles (95°C 30 sec, 56°C 30 sec and 70°C 30 sec), 72°C 5 min
CG12232308	IER3	Hs_CG12232308_01_PM	244	Reverse primer	chr6:30711398-30711642	6	95°C 6min,45 cycles (95°C 30 sec, 62°C 30 sec and 70°C 30 sec), 72°C 5 min
CG11664500	EYA4	Hs_CG11664500_01_PM	249	Reverse primer	chr6:133562278-133562527	9	95°C 6min,45 cycles (95°C 30 sec, 58°C 30 sec and 70°C 30 sec), 72°C 5 min
CG22903300	HCG4	Hs_CG22903300_03_PM	209	Reverse primer	chr6:29760652-29760861	7	95°C 6min,45 cycles (95°C 30 sec, 56°C 30 sec and 70°C 30 sec), 72°C 5 min

Supplementary Table S4: KEGG pathways associated with *de novo* methylation in NPC

Term	Count	%	p value	Fold Enrichment	Adjusted p value
hsa04020:Calcium signaling pathway	44	2.38	4.42E-08	2.39	7.52E-06
hsa04950:Maturity onset diabetes of the young	14	0.76	2.72E-07	5.30	2.31E-05
hsa05200:Pathways in cancer	55	2.97	3.57E-04	1.60	2.00E-02
hsa05217:Basal cell carcinoma	16	0.86	3.93E-04	2.75	1.66E-02
hsa04310:Wnt signaling pathway	30	1.62	7.97E-04	1.89	2.67E-02
hsa04510:Focal adhesion	36	1.95	0.001441	1.71	4.00E-02
hsa04080:Neuroactive ligand-receptor interaction	43	2.32	0.001515	1.61	3.62E-02
hsa04070:Phosphatidylinositol signaling system	17	0.92	0.00373	2.17	7.63E-02
hsa04810:Regulation of actin cytoskeleton	36	1.95	0.004309	1.61	7.83E-02
hsa04916:Melanogenesis	20	1.08	0.006665	1.91	1.07E-01
hsa04010:MAPK signaling pathway	41	2.22	0.012736	1.45	1.80E-01
hsa04350:TGF-beta signaling pathway	17	0.92	0.016332	1.87	2.08E-01
hsa05412:Arrhythmogenic right ventricular cardiomyopathy (ARVC)	15	0.81	0.020778	1.92	2.40E-01
hsa04540:Gap junction	17	0.92	0.02219	1.81	2.39E-01
hsa04360:Axon guidance	22	1.19	0.027704	1.61	2.73E-01
hsa04340:Hedgehog signaling pathway	12	0.65	0.029594	2.03	2.73E-01
hsa04670:Leukocyte transendothelial migration	20	1.08	0.03067	1.65	2.68E-01

Supplementary Table S5: *De Novo* **methylated regions in NPC**

Index	Chr	start	end	Gene	Region	length	β_{PBMC}	β_{normal}	β_{tumour}	# CG site	correct FDR	H3K27me3	H3K4me3
1	6	33245460	33245638	B3GALT4	chr6:33245460-33245638	178	0.14	0.14	0.26	12	3.83E-24	0	1
2	6	30228003	30228083	HLA-L	chr6:30228003-30228083	80	0.07	0.12	0.39	8	5.12E-20	1	1
3	6	32116841	32116994	PRRT1	chr6:32116841-32116994	153	0.08	0.21	0.32	11	1.31E-19	1	1
4	12	115136175	115136308		chr12:115136175-115136308	133	0.07	0.15	0.32	9	1.31E-19	1	1
5	12	115134886	115135076		chr12:115134886-115135076	190	0.08	0.20	0.38	12	7.00E-19	1	0
6	6	32055370	32055534	TNXB	chr6:32055370-32055534	164	0.05	0.07	0.24	9	8.34E-18	1	1
7	5	33936232	33936402	RXFP3	chr5:33936232-33936402	170	0.03	0.11	0.30	8	6.00E-17	1	1
8	7	50861418	50861654	GRB10	chr7:50861418-50861654	236	0.10	0.20	0.33	10	1.45E-16	1	1
9	6	133562461	133562494	EYA4	chr6:133562461-133562494	33	0.05	0.19	0.38	9	4.49E-16	1	1
10	2	25391656	25391720	POMC	chr2:25391656-25391720	64	0.12	0.14	0.38	6	1.27E-15	1	1
11	6	152128328	152128588	ESR1	chr6:152128328-152128588	260	0.09	0.12	0.18	13	2.04E-15	1	1
12	7	94284380	94284539	SGCE;PEG10	chr7:94284380-94284539	159	0.06	0.07	0.23	11	7.25E-15	0	1
13	6	28956226	28956327		chr6:28956226-28956327	101	0.12	0.16	0.38	9	4.67E-14	0	1
14	1	3568210	3568245	TP73	chr1:3568210-3568245	35	0.05	0.24	0.48	5	5.20E-14	1	1
15	4	25657365	25657440	SLC34A2	chr4:25657365-25657440	75	0.06	0.23	0.45	6	8.02E-14	1	1
16	11	6341842	6341908	PRKCDBP	chr11:6341842-6341908	66	0.08	0.10	0.27	7	8.13E-14	0	1
17	11	43602845	43602965	MIR129-2	chr11:43602845-43602965	120	0.03	0.11	0.31	7	9.11E-14	1	1
18	19	9473674	9473715	ZNF177	chr19:9473674-9473715	41	0.09	0.30	0.45	6	2.24E-13	0	0
19	5	16180033	16180076	MARCH11	chr5:16180033-16180076	43	0.03	0.21	0.39	7	2.86E-13	1	1
20	2	24232847	24232891	MFSD2B	chr2:24232847-24232891	44	0.03	0.04	0.16	7	6.44E-13	1	1
21	18	74961724	74961809	GALR1	chr18:74961724-74961809	85	0.07	0.22	0.38	7	7.25E-13	1	1
22	19	11784647	11784774	ZNF833	chr19:11784647-11784774	127	0.10	0.14	0.31	6	1.22E-12	0	1
23	2	182521874	182521926	CERKL	chr2:182521874-182521926	52	0.04	0.04	0.15	6	1.93E-12	1	1
24	6	30698706	30698784	FLOT1	chr6:30698706-30698784	78	0.08	0.16	0.27	6	2.00E-12	0	0
25	3	13590415	13590450	FBLN2	chr3:13590415-13590450	35	0.16	0.27	0.41	6	2.42E-12	1	1
26	6	166582159	166582206	T	chr6:166582159-166582206	47	0.05	0.14	0.38	5	7.24E-12	1	1
27	19	21106002	21106053	ZNF85	chr19:21106002-21106053	51	0.08	0.16	0.30	6	9.34E-12	0	1
28	19	17982682	17982759	SLC5A5	chr19:17982682-17982759	77	0.07	0.09	0.16	6	9.56E-12	0	0
29	2	201450506	201450633	AOX1	chr2:201450506-201450633	127	0.04	0.06	0.13	7	1.01E-11	1	1
30	10	134901279	134901305	GPR123	chr10:134901279-134901305	26	0.03	0.22	0.38	5	1.03E-11	1	1
31	10	134600651	134600701	NKX6-2	chr10:134600651-134600701	50	0.02	0.21	0.38	5	1.39E-11	1	0
32	7	105221985	105222048	EFCAB10	chr7:105221985-105222048	63	0.06	0.07	0.28	6	1.48E-11	0	1
33	6	28602566	28602791		chr6:28602566-28602791	225	0.09	0.11	0.19	11	1.79E-11	1	1

34	20	43729861	43729910	KCNS1	chr20:43729861-43729910	49	0.27	0.25	0.43	5	1.96E-11	1	1
35	4	76555772	76555856	CDKL2	chr4:76555772-76555856	84	0.04	0.05	0.20	6	2.02E-11	0	1
36	7	27205114	27205230	HOXA9	chr7:27205114-27205230	116	0.03	0.11	0.26	6	2.07E-11	1	0
37	16	66613206	66613278	CMTM2	chr16:66613206-66613278	72	0.03	0.22	0.33	5	3.07E-11	1	1
38	6	30071344	30071427	TRIM31	chr6:30071344-30071427	83	0.06	0.08	0.16	8	3.14E-11	1	1
39	8	38964944	38965026	ADAM32	chr8:38964944-38965026	82	0.09	0.21	0.43	5	3.67E-11	0	0
40	18	60988017	60988096	BCL2	chr18:60988017-60988096	79	0.08	0.08	0.25	5	3.70E-11	1	1
41	6	28557388	28557575		chr6:28557388-28557575	187	0.08	0.07	0.17	9	4.67E-11	0	1
42	15	90039794	90039822	RHCG	chr15:90039794-90039822	28	0.08	0.14	0.37	5	5.38E-11	1	1
43	12	111284768	111284826	CCDC63	chr12:111284768-111284826	58	0.02	0.06	0.35	5	7.09E-11	0	1
44	20	36793705	36793747	TGM2	chr20:36793705-36793747	42	0.03	0.05	0.39	5	7.92E-11	1	1
45	7	30029717	30029808	SCRN1	chr7:30029717-30029808	91	0.14	0.20	0.32	6	1.08E-10	0	1
46	17	73584029	73584111	MYO15B	chr17:73584029-73584111	82	0.08	0.09	0.26	5	1.26E-10	1	1
47	22	45680676	45680702	UPK3A	chr22:45680676-45680702	26	0.04	0.09	0.37	4	1.60E-10	1	1
48	17	16256937	16257010	CENPV	chr17:16256937-16257010	73	0.03	0.03	0.17	5	1.78E-10	0	1
49	8	1765296	1765387	MIR596	chr8:1765296-1765387	91	0.05	0.14	0.24	6	1.82E-10	0	1
50	12	8332789	8332810	FAM66C	chr12:8332789-8332810	21	0.05	0.07	0.19	4	2.72E-10	0	0
51	12	54070517	54070610	ATP5G2	chr12:54070517-54070610	93	0.12	0.12	0.33	5	3.04E-10	0	1
52	13	50070436	50070550	PHF11	chr13:50070436-50070550	114	0.02	0.06	0.27	6	3.41E-10	0	1
53	10	97802912	97802977	CCNJ	chr10:97802912-97802977	65	0.12	0.17	0.31	6	4.21E-10	0	1
54	5	156886970	156887005	NIPAL4	chr5:156886970-156887005	35	0.07	0.09	0.20	5	4.88E-10	1	1
55	20	21686251	21686308	PAX1	chr20:21686251-21686308	57	0.04	0.21	0.33	7	6.52E-10	1	0
56	7	101006019	101006089	EMID2	chr7:101006019-101006089	70	0.07	0.24	0.43	6	7.69E-10	0	1
57	12	133464855	133464933	CHFR	chr12:133464855-133464933	78	0.19	0.17	0.30	5	9.10E-10	0	1
58	1	31845904	31845985	FABP3	chr1:31845904-31845985	81	0.11	0.15	0.28	5	9.56E-10	0	1
59	2	85980731	85980803	ATOH8	chr2:85980731-85980803	72	0.04	0.05	0.15	6	9.60E-10	1	1
60	6	30227783	30227883	HLA-L	chr6:30227783-30227883	100	0.05	0.12	0.25	7	1.08E-09	1	1
61	20	42544648	42544666	TOX2	chr20:42544648-42544666	18	0.15	0.17	0.40	4	1.10E-09	1	1
62	2	66667039	66667101	MEIS1	chr2:66667039-66667101	62	0.08	0.15	0.37	5	1.12E-09	1	1
63	3	136537694	136537863	TMEM22	chr3:136537694-136537863	169	0.05	0.08	0.23	8	1.19E-09	1	1
64	8	65489863	65489939	LOC401463	chr8:65489863-65489939	76	0.08	0.12	0.28	5	1.51E-09	1	1
65	20	37353096	37353158	SLC32A1	chr20:37353096-37353158	62	0.03	0.16	0.32	4	1.60E-09	1	1
66	8	23563925	23564031	NKX2-6	chr8:23563925-23564031	106	0.04	0.26	0.39	5	1.76E-09	1	1
67	19	20278013	20278059	ZNF486	chr19:20278013-20278059	46	0.04	0.10	0.21	4	2.01E-09	0	1
68	11	64739320	64739374		chr11:64739320-64739374	54	0.18	0.18	0.41	4	2.06E-09	1	0

69	15	26108391	26108412	ATP10A	chr15:26108391-26108412	21	0.02	0.15	0.37	5	2.22E-09	1	1
70	6	28227068	28227093	NKAPL	chr6:28227068-28227093	25	0.16	0.29	0.40	5	2.26E-09	1	0
71	10	11206772	11206892	CUGBP2	chr10:11206772-11206892	120	0.06	0.03	0.07	7	2.43E-09	0	1
72	19	56879554	56879613	ZNF542	chr19:56879554-56879613	59	0.03	0.10	0.33	4	3.05E-09	0	1
73	20	13200929	13200992	ISM1	chr20:13200929-13200992	63	0.03	0.30	0.39	10	3.24E-09	1	1
74	20	25129507	25129562	LOC284798	chr20:25129507-25129562	55	0.05	0.15	0.26	5	3.60E-09	1	1
75	6	31649432	31649519	LY6G5C	chr6:31649432-31649519	87	0.11	0.13	0.24	5	3.79E-09	1	1
76	3	32859377	32859445	TRIM71	chr3:32859377-32859445	68	0.04	0.09	0.21	5	3.93E-09	0	1
77	12	2800446	2800500	CACNA1C	chr12:2800446-2800500	54	0.04	0.07	0.20	4	4.20E-09	1	1
78	20	25062754	25062860	VSX1	chr20:25062754-25062860	106	0.10	0.24	0.33	7	4.24E-09	1	1
79	11	2160875	2161009	IGF2AS;IGF2	chr11:2160875-2161009	134	0.09	0.19	0.27	8	4.71E-09	1	0
80	18	74961424	74961494	GALR1	chr18:74961424-74961494	70	0.07	0.14	0.25	5	4.95E-09	1	1
81	1	9884565	9884624	CLSTN1	chr1:9884565-9884624	59	0.05	0.13	0.23	6	4.97E-09	1	1
82	15	96868935	96869043	NR2F2	chr15:96868935-96869043	108	0.09	0.12	0.23	5	5.03E-09	1	0
83	6	30647034	30647128	KIAA1949	chr6:30647034-30647128	94	0.12	0.10	0.34	4	5.10E-09	0	1
84	11	17373103	17373180	DKFZp686O24166	chr11:17373103-17373180	77	0.04	0.04	0.11	5	5.14E-09	1	1
85	5	140810106	140810161	PCDHGA4	chr5:140810106-140810161	55	0.20	0.24	0.42	5	5.46E-09	0	0
86	6	166722053	166722062	PRR18	chr6:166722053-166722062	9	0.13	0.19	0.29	4	5.70E-09	1	1
87	11	44332624	44332838	ALX4	chr11:44332624-44332838	214	0.12	0.20	0.30	10	5.91E-09	1	1
88	16	1030561	1030619	SOX8	chr16:1030561-1030619	58	0.02	0.21	0.42	4	6.06E-09	1	1
89	21	34398199	34398265	OLIG2	chr21:34398199-34398265	66	0.03	0.22	0.39	5	6.71E-09	1	1
90	17	48546258	48546321	CHAD;ACSF2	chr17:48546258-48546321	63	0.06	0.28	0.47	4	6.75E-09	1	1
91	17	42733662	42733729	C17orf104	chr17:42733662-42733729	67	0.09	0.18	0.37	4	7.12E-09	1	0
92	12	8025567	8025640	SLC2A14	chr12:8025567-8025640	73	0.11	0.23	0.39	4	7.14E-09	0	1
93	7	150102721	150102791	LOC728743	chr7:150102721-150102791	70	0.04	0.02	0.20	4	7.67E-09	1	1
94	15	55880997	55881081	PYGO1	chr15:55880997-55881081	84	0.06	0.15	0.33	4	8.03E-09	1	1
95	1	165414491	165414611	RXRG	chr1:165414491-165414611	120	0.08	0.15	0.28	6	8.94E-09	1	1
96	15	26108612	26108683	ATP10A	chr15:26108612-26108683	71	0.04	0.07	0.18	5	9.33E-09	1	1
97	12	26986763	26986864	ITPR2	chr12:26986763-26986864	101	0.04	0.03	0.14	5	1.06E-08	0	1
98	17	41924466	41924523	CD300LG	chr17:41924466-41924523	57	0.12	0.11	0.26	5	1.11E-08	0	1
99	2	106681937	106681998	C2orf40	chr2:106681937-106681998	61	0.10	0.26	0.39	4	1.12E-08	1	1
100	10	123923050	123923099	TACC2	chr10:123923050-123923099	49	0.06	0.12	0.32	4	1.30E-08	0	1
101	6	133562246	133562275	EYA4	chr6:133562246-133562275	29	0.06	0.12	0.30	5	1.31E-08	1	1
102	2	31361757	31361818	GALNT14	chr2:31361757-31361818	61	0.13	0.14	0.26	4	1.52E-08	1	1

103	1	179545073	179545166	NPHS2	chr1:179545073-179545166	93	0.04	0.18	0.30	5	1.59E-08	1	1
104	10	100993553	100993597	HPSE2	chr10:100993553-100993597	44	0.05	0.13	0.29	6	1.67E-08	1	1
105	1	58715499	58715553	DAB1	chr1:58715499-58715553	54	0.04	0.10	0.33	4	1.70E-08	1	1
106	19	44324856	44324951	LYPD5	chr19:44324856-44324951	95	0.10	0.20	0.32	6	1.82E-08	0	0
107	12	115135859	115135934		chr12:115135859-115135934	75	0.12	0.19	0.33	4	1.87E-08	1	1
108	17	46799948	46800028	PRAC;C17orf93	chr17:46799948-46800028	80	0.12	0.19	0.37	4	1.88E-08	1	1
109	1	178693969	178694013	RALGPS2	chr1:178693969-178694013	44	0.08	0.08	0.25	4	1.99E-08	0	1
110	6	36410485	36410553	PXT1;KCTD20	chr6:36410485-36410553	68	0.03	0.03	0.04	7	2.05E-08	0	1
111	4	144621898	144621971	FREM3	chr4:144621898-144621971	73	0.04	0.12	0.25	5	2.14E-08	1	1
112	11	111783327	111783355	CRYAB;HSPB2	chr11:111783327-111783355	28	0.22	0.25	0.39	4	2.19E-08	0	0
113	10	102891019	102891080	TLX1NB;TLX1	chr10:102891019-102891080	61	0.04	0.15	0.35	4	2.35E-08	1	0
114	3	50378407	50378431	RASSF1	chr3:50378407-50378431	24	0.05	0.03	0.17	5	2.69E-08	1	1
115	2	73429308	73429371	NOTO	chr2:73429308-73429371	63	0.08	0.18	0.32	5	2.88E-08	1	1
116	5	87974369	87974464	LOC645323	chr5:87974369-87974464	95	0.12	0.21	0.31	6	2.89E-08	1	1
117	6	28510281	28510376		chr6:28510281-28510376	95	0.06	0.11	0.20	7	3.04E-08	0	1
118	11	86383236	86383300	ME3	chr11:86383236-86383300	64	0.04	0.15	0.39	5	3.13E-08	1	1
119	19	38747355	38747378	PPP1R14A	chr19:38747355-38747378	23	0.05	0.22	0.47	3	3.32E-08	1	0
120	3	46506481	46506554	LTF	chr3:46506481-46506554	73	0.17	0.23	0.35	5	3.35E-08	1	1
121	20	37434229	37434262	PPP1R16B	chr20:37434229-37434262	33	0.02	0.06	0.32	4	3.38E-08	1	1
122	11	2161079	2161141	IGF2AS;IGF2	chr11:2161079-2161141	62	0.07	0.18	0.28	6	3.57E-08	1	0
123	6	2972158	2972195	SERPINB6	chr6:2972158-2972195	37	0.12	0.13	0.26	5	3.80E-08	1	1
124	7	127670993	127671044	LRRC4;SND1	chr7:127670993-127671044	51	0.16	0.26	0.40	4	4.01E-08	0	1
125	20	61809827	61809841	MIR124-3	chr20:61809827-61809841	14	0.07	0.16	0.26	4	4.17E-08	1	1
126	2	29338100	29338121	CLIP4	chr2:29338100-29338121	21	0.04	0.06	0.15	4	4.40E-08	1	1
127	19	56904945	56905032	ZNF582	chr19:56904945-56905032	87	0.04	0.14	0.30	6	4.40E-08	0	1
128	13	20768523	20768560	GJB2	chr13:20768523-20768560	37	0.07	0.06	0.22	3	4.52E-08	1	1
129	17	72733163	72733183	RAB37	chr17:72733163-72733183	20	0.02	0.04	0.23	4	4.64E-08	1	1
130	14	59932101	59932153	GPR135	chr14:59932101-59932153	52	0.02	0.06	0.16	5	4.84E-08	1	1
131	6	169653905	169653961	THBS2	chr6:169653905-169653961	56	0.12	0.24	0.40	5	5.24E-08	1	1
132	2	85107426	85107488	C2orf89	chr2:85107426-85107488	62	0.07	0.05	0.32	3	5.25E-08	1	1
133	19	38747174	38747234	PPP1R14A	chr19:38747174-38747234	60	0.04	0.12	0.37	3	5.26E-08	1	1
134	12	15374513	15374609	RERG	chr12:15374513-15374609	96	0.05	0.04	0.19	4	5.28E-08	1	0
135	6	32119616	32119691	PRRT1	chr6:32119616-32119691	75	0.16	0.28	0.54	4	5.47E-08	1	1
136	4	165304443	165304540	MARCH1	chr4:165304443-165304540	97	0.12	0.23	0.34	6	5.58E-08	1	1
137	18	30051521	30051572	FAM59A	chr18:30051521-30051572	51	0.05	0.12	0.26	5	5.69E-08	1	1

138	7	27225733	27225897	HOXA11AS;HOXA11	chr7:27225733-27225897	164	0.20	0.21	0.36	6	5.92E-08	1	1
139	12	115136060	115136098		chr12:115136060-115136098	38	0.07	0.17	0.37	4	6.07E-08	1	1
140	11	133907726	133907750	LOC100128239	chr11:133907726-133907750	24	0.05	0.03	0.13	3	6.21E-08	1	1
141	13	37006063	37006133	CCNA1	chr13:37006063-37006133	70	0.06	0.10	0.25	5	6.54E-08	1	1
142	1	13910138	13910224	PDPN	chr1:13910138-13910224	86	0.08	0.12	0.22	6	6.74E-08	0	1
143	5	1876168	1876215		chr5:1876168-1876215	47	0.11	0.12	0.32	3	7.03E-08	1	1
144	7	19158647	19158692	TWIST1	chr7:19158647-19158692	45	0.05	0.19	0.39	4	7.05E-08	1	0
145	6	30095546	30095570		chr6:30095546-30095570	24	0.08	0.10	0.31	3	8.30E-08	1	1
146	19	57019005	57019069	ZNF471	chr19:57019005-57019069	64	0.04	0.30	0.49	4	8.68E-08	0	1
147	1	38470540	38470665	FHL3	chr1:38470540-38470665	125	0.07	0.09	0.23	4	8.73E-08	0	1
148	16	3068315	3068382	CLDN6	chr16:3068315-3068382	67	0.04	0.08	0.17	4	8.93E-08	1	1
149	13	78493282	78493365	EDNRB	chr13:78493282-78493365	83	0.04	0.12	0.21	7	8.99E-08	0	1
150	12	133065906	133065941	FBRSL1	chr12:133065906-133065941	35	0.03	0.06	0.15	6	9.40E-08	1	1
151	2	37571677	37571732	QPCT	chr2:37571677-37571732	55	0.08	0.15	0.28	5	9.56E-08	1	1
152	4	42399843	42399858	SHISA3	chr4:42399843-42399858	15	0.02	0.06	0.43	3	1.00E-07	1	1
153	13	112721712	112721789	SOX1	chr13:112721712-112721789	77	0.13	0.28	0.35	6	1.04E-07	1	1
154	6	30523350	30523440	GNL1;PRR3	chr6:30523350-30523440	90	0.05	0.04	0.09	6	1.05E-07	0	1
155	17	43339476	43339515	C17orf46	chr17:43339476-43339515	39	0.03	0.05	0.17	4	1.07E-07	0	1
156	18	30051176	30051374	FAM59A	chr18:30051176-30051374	198	0.05	0.06	0.10	10	1.08E-07	1	1
157	3	141516232	141516291	GRK7	chr3:141516232-141516291	59	0.03	0.04	0.32	3	1.10E-07	1	1
158	12	54409207	54409257	HOXC4;HOXC6;HOXC5	chr12:54409207-54409257	50	0.12	0.25	0.45	4	1.13E-07	1	0
159	6	105628000	105628063	POPDC3	chr6:105628000-105628063	63	0.04	0.12	0.20	7	1.20E-07	1	1
160	2	39187533	39187543	LOC375196	chr2:39187533-39187543	10	0.01	0.10	0.30	3	1.21E-07	1	1
161	10	82116365	82116456	DYDC2;DYDC1	chr10:82116365-82116456	91	0.05	0.13	0.24	6	1.21E-07	0	1
162	1	45792660	45792733	HPDL	chr1:45792660-45792733	73	0.02	0.03	0.14	4	1.26E-07	0	1
163	6	117086996	117087083	FAM162B	chr6:117086996-117087083	87	0.10	0.16	0.26	4	1.29E-07	1	1
164	1	92952440	92952467	GFI1	chr1:92952440-92952467	27	0.06	0.12	0.42	3	1.30E-07	1	1
165	6	133562035	133562101	EYA4	chr6:133562035-133562101	66	0.07	0.13	0.26	4	1.30E-07	1	1
166	14	52535949	52536022	NID2	chr14:52535949-52536022	73	0.08	0.13	0.26	5	1.30E-07	1	1
167	1	164290179	164290275		chr1:164290179-164290275	96	0.18	0.24	0.45	4	1.31E-07	0	0
168	2	176971961	176972113	HOXD11	chr2:176971961-176972113	152	0.11	0.22	0.31	7	1.37E-07	1	1
169	8	37595307	37595363	ERLIN2	chr8:37595307-37595363	56	0.04	0.01	0.09	3	1.39E-07	0	1
170	11	131780329	131780357	NTM	chr11:131780329-131780357	28	0.06	0.20	0.31	5	1.43E-07	1	1
171	3	121902561	121902622	CASR	chr3:121902561-121902622	61	0.22	0.27	0.46	3	1.54E-07	1	1

172	1	236849942	236849994	ACTN2	chr1:236849942-236849994	52	0.05	0.25	0.44	3	1.55E-07	1	1
173	2	87088796	87088843	CD8B	chr2:87088796-87088843	47	0.17	0.24	0.38	4	1.57E-07	1	1
174	6	30095728	30095802		chr6:30095728-30095802	74	0.10	0.09	0.26	3	1.62E-07	1	1
175	17	66255210	66255265	ARSG	chr17:66255210-66255265	55	0.05	0.03	0.13	4	1.63E-07	0	0
176	6	110797476	110797506	SLC22A16	chr6:110797476-110797506	30	0.05	0.08	0.36	3	1.65E-07	0	0
177	7	94284623	94284678	SGCE;PEG10	chr7:94284623-94284678	55	0.06	0.05	0.20	4	1.66E-07	0	1
178	17	5026540	5026574	ZNF232	chr17:5026540-5026574	34	0.06	0.09	0.20	3	1.67E-07	0	0
179	1	248100585	248100614	OR2L13	chr1:248100585-248100614	29	0.23	0.22	0.34	4	1.75E-07	0	0
180	14	100111263	100111354	HHIPL1	chr14:100111263-100111354	91	0.11	0.27	0.36	6	2.06E-07	1	1
181	12	54071165	54071194	ATP5G2	chr12:54071165-54071194	29	0.12	0.22	0.48	3	2.06E-07	0	1
182	19	53073242	53073334	ZNF701	chr19:53073242-53073334	92	0.13	0.21	0.40	4	2.08E-07	0	1
183	10	15761854	15761881	ITGA8	chr10:15761854-15761881	27	0.06	0.19	0.30	4	2.10E-07	1	1
184	5	41870856	41870910	OXCT1	chr5:41870856-41870910	54	0.06	0.03	0.09	5	2.14E-07	1	1
185	1	67772972	67773044	IL12RB2	chr1:67772972-67773044	72	0.08	0.22	0.37	4	2.19E-07	1	1
186	12	4384879	4384890	CCND2	chr12:4384879-4384890	11	0.06	0.07	0.26	3	2.19E-07	1	1
187	15	101419243	101419333	ALDH1A3	chr15:101419243-101419333	90	0.08	0.21	0.47	7	2.20E-07	1	1
188	10	135050326	135050355	VENTX	chr10:135050326-135050355	29	0.04	0.28	0.43	4	2.26E-07	1	1
189	12	113909932	113910034	LHX5	chr12:113909932-113910034	102	0.11	0.15	0.20	6	2.29E-07	1	1
190	15	79383841	79383890	RASGRF1	chr15:79383841-79383890	49	0.06	0.05	0.13	5	2.35E-07	1	1
191	5	5139853	5139878	ADAMTS16	chr5:5139853-5139878	25	0.05	0.24	0.40	5	2.37E-07	1	1
192	2	239140169	239140190	LOC151174	chr2:239140169-239140190	21	0.02	0.12	0.43	3	2.40E-07	1	1
193	3	45838028	45838094	SLC6A20	chr3:45838028-45838094	66	0.04	0.09	0.18	5	2.41E-07	1	1
194	12	115134344	115134391		chr12:115134344-115134391	47	0.16	0.26	0.43	4	2.45E-07	1	0
195	22	31002942	31003010	TCN2	chr22:31002942-31003010	68	0.08	0.10	0.21	4	2.46E-07	1	1
196	1	150947566	150947589	LASS2	chr1:150947566-150947589	23	0.04	0.04	0.29	3	2.50E-07	0	1
197	8	132052843	132052942	ADCY8	chr8:132052843-132052942	99	0.08	0.21	0.31	5	2.59E-07	1	1
198	20	61051751	61051802	GATA5	chr20:61051751-61051802	51	0.09	0.12	0.24	5	2.62E-07	1	0
199	20	56725833	56725899	C20orf85	chr20:56725833-56725899	66	0.09	0.15	0.23	6	2.69E-07	1	1
200	8	61194072	61194111	CA8	chr8:61194072-61194111	39	0.02	0.06	0.19	5	2.80E-07	0	1
201	19	58220773	58220837	ZNF154	chr19:58220773-58220837	64	0.14	0.23	0.42	3	2.85E-07	0	1
202	22	23522460	23522551	BCR	chr22:23522460-23522551	91	0.08	0.09	0.21	5	2.96E-07	1	1
203	6	30228153	30228169	HLA-L	chr6:30228153-30228169	16	0.25	0.23	0.44	3	2.98E-07	1	1
204	12	123380870	123380891	VPS37B	chr12:123380870-123380891	21	0.17	0.25	0.41	4	3.02E-07	1	1
205	7	149569946	149570071	ATP6V0E2	chr7:149569946-149570071	125	0.20	0.22	0.31	6	3.03E-07	1	1
206	10	25464132	25464196	LOC100128811;GPR158	chr10:25464132-25464196	64	0.08	0.26	0.34	5	3.04E-07	1	1

207	1	236228741	236228755	NID1	chr1:236228741-236228755	14	0.04	0.27	0.42	3	3.12E-07	1	1
208	12	3862417	3862497	EFCAB4B	chr12:3862417-3862497	80	0.04	0.05	0.13	7	3.15E-07	1	1
209	18	904851	904963	ADCYAP1	chr18:904851-904963	112	0.07	0.18	0.25	6	3.41E-07	1	1
210	19	22605151	22605234	ZNF98	chr19:22605151-22605234	83	0.11	0.24	0.36	4	3.63E-07	0	0
211	1	154377364	154377429	IL6R	chr1:154377364-154377429	65	0.11	0.17	0.29	4	3.76E-07	1	1
212	1	244213577	244213618	ZNF238	chr1:244213577-244213618	41	0.13	0.20	0.47	3	3.85E-07	1	1
213	10	50887566	50887632	C10orf53	chr10:50887566-50887632	66	0.08	0.16	0.26	5	3.88E-07	1	1
214	22	23522307	23522374	BCR;BCR	chr22:23522307-23522374	67	0.25	0.27	0.46	3	4.01E-07	1	1
215	19	22235256	22235281	ZNF257	chr19:22235256-22235281	25	0.19	0.27	0.40	3	4.04E-07	0	0
216	10	28034792	28034856	MKX	chr10:28034792-28034856	64	0.04	0.29	0.39	6	4.12E-07	1	1
217	1	41444788	41444871	CTPS	chr1:41444788-41444871	83	0.25	0.26	0.43	6	4.31E-07	0	1
218	1	111217691	111217715	KCNA3	chr1:111217691-111217715	24	0.05	0.06	0.33	3	4.39E-07	1	1
219	10	71333152	71333216	NEUROG3	chr10:71333152-71333216	64	0.06	0.08	0.13	5	4.59E-07	1	1
220	21	44073202	44073246	PDE9A	chr21:44073202-44073246	44	0.21	0.19	0.36	3	4.76E-07	1	1
221	19	15580383	15580445	PGLYRP2	chr19:15580383-15580445	62	0.10	0.19	0.42	3	4.77E-07	1	0
222	8	67344553	67344588	ADHFE1	chr8:67344553-67344588	35	0.04	0.10	0.45	3	4.78E-07	0	1
223	16	30075511	30075547	ALDOA	chr16:30075511-30075547	36	0.04	0.04	0.27	3	5.10E-07	0	1
224	17	45810359	45810436	TBX21	chr17:45810359-45810436	77	0.20	0.12	0.24	6	5.19E-07	1	1
225	22	44287841	44287958	PNPLA5	chr22:44287841-44287958	117	0.10	0.11	0.18	5	5.22E-07	1	1
226	14	24641155	24641189	REC8	chr14:24641155-24641189	34	0.08	0.19	0.32	3	5.27E-07	0	1
227	2	29337946	29337988	CLIP4	chr2:29337946-29337988	42	0.10	0.07	0.20	3	5.45E-07	1	1
228	20	21494679	21494762	NKX2-2	chr20:21494679-21494762	83	0.15	0.29	0.38	6	5.46E-07	1	1
229	6	30711636	30711680	IER3;FLOT1	chr6:30711636-30711680	44	0.04	0.03	0.12	5	5.49E-07	0	1
230	15	74658547	74658573	CYP11A1	chr15:74658547-74658573	26	0.03	0.07	0.38	3	5.52E-07	1	1
231	1	6515580	6515624	ESPN	chr1:6515580-6515624	44	0.14	0.25	0.47	3	5.58E-07	1	0
232	12	119772354	119772421	CCDC60	chr12:119772354-119772421	67	0.17	0.19	0.33	4	5.58E-07	1	1
233	11	111385450	111385496	C11orf88	chr11:111385450-111385496	46	0.03	0.07	0.28	3	5.60E-07	1	1
234	5	40835740	40835760	RPL37	chr5:40835740-40835760	20	0.19	0.11	0.28	3	5.93E-07	0	1
235	8	143858612	143858636	LYNX1	chr8:143858612-143858636	24	0.05	0.26	0.43	3	6.14E-07	0	1
236	3	137483479	137483555	SOX14	chr3:137483479-137483555	76	0.17	0.27	0.36	5	6.36E-07	1	1
237	12	4381777	4381803	CCND2	chr12:4381777-4381803	26	0.03	0.01	0.04	5	6.37E-07	1	1
238	12	104697387	104697419	TXNRD1	chr12:104697387-104697419	32	0.13	0.16	0.40	3	6.47E-07	0	1
239	6	151814892	151814994	C6orf97	chr6:151814892-151814994	102	0.17	0.16	0.24	5	6.55E-07	1	1
240	4	188916668	188916726	ZFP42	chr4:188916668-188916726	58	0.08	0.29	0.44	4	6.76E-07	0	1

241	4	48485289	48485328	SLC10A4	chr4:48485289-48485328	39	0.08	0.15	0.24	5	6.86E-07	1	1
242	11	105481283	105481322	GRIA4	chr11:105481283-105481322	39	0.04	0.15	0.27	6	6.91E-07	1	1
243	4	2765896	2765951		chr4:2765896-2765951	55	0.04	0.02	0.15	3	7.21E-07	0	1
244	6	87646922	87646986	HTR1E	chr6:87646922-87646986	64	0.11	0.11	0.22	4	7.33E-07	1	1
245	11	7041491	7041685	ZNF214;NLRP14	chr11:7041491-7041685	194	0.05	0.10	0.16	9	7.45E-07	0	1
246	6	26240505	26240528	HIST1H4F	chr6:26240505-26240528	23	0.05	0.05	0.18	3	7.52E-07	0	1
247	6	62996119	62996132	KHDRBS2	chr6:62996119-62996132	13	0.05	0.16	0.36	3	7.63E-07	0	1
248	14	69726197	69726254	GALNTL1	chr14:69726197-69726254	57	0.07	0.18	0.29	4	7.76E-07	1	1
249	7	3083721	3083773	CARD11	chr7:3083721-3083773	52	0.10	0.07	0.29	3	7.95E-07	0	1
250	2	218621486	218621497	DIRC3	chr2:218621486-218621497	11	0.23	0.20	0.42	3	8.29E-07	1	0
251	1	24882153	24882207	C1orf130	chr1:24882153-24882207	54	0.12	0.25	0.43	3	8.31E-07	1	1
252	8	67344640	67344665	ADHFE1	chr8:67344640-67344665	25	0.07	0.13	0.40	3	8.40E-07	0	1
253	1	207627492	207627601	CR2	chr1:207627492-207627601	109	0.04	0.03	0.08	4	8.44E-07	1	1
254	10	47083283	47083355	PPYR1	chr10:47083283-47083355	72	0.16	0.16	0.30	4	8.51E-07	1	1
255	2	175208588	175208605		chr2:175208588-175208605	17	0.06	0.16	0.42	3	8.51E-07	1	1
256	3	26663551	26663626	LRRC3B	chr3:26663551-26663626	75	0.20	0.30	0.43	3	8.55E-07	1	1

Supplementary Method: region-specific methylation analysis on HumanMethylation450 BeadChip

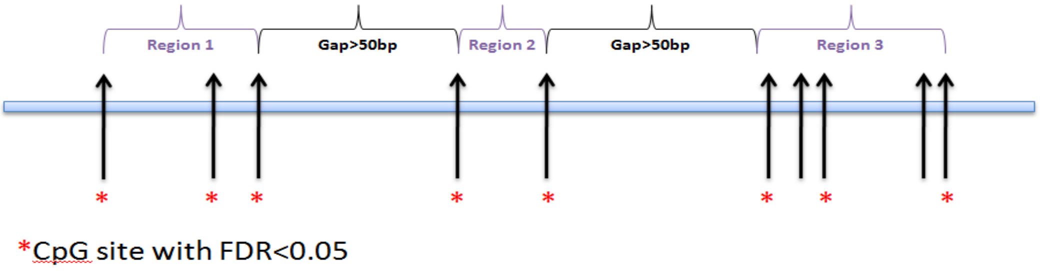

$$FDR_{region} = 10^{2k} \prod_{i=1}^{k} FDR_i$$ **Equation (1)**

As shown in the figure above, the adjacent CpG sites within 50bp were merged into one region. So we integrated the individual CpG sites targeted by HumanMethylation450 into small regions. The significance level of differential methylation for a region is estimated as Equation (1), where k is the number of CpG sites with FDR<0.05 from LIMMA analysis within the region. To avoid the array-design bias that the genomic regions with high density of genes have better coverage by HumanMethylation450 BeaChip than the poor-gene regions, we corrected FDR of a region using a correction parameter 10^{2k}, thus the number of CpG sites designed on the BeadChip within the region was taken into consideration in the analysis.

Supplementary Figure S1 Power estimation in the discovery set

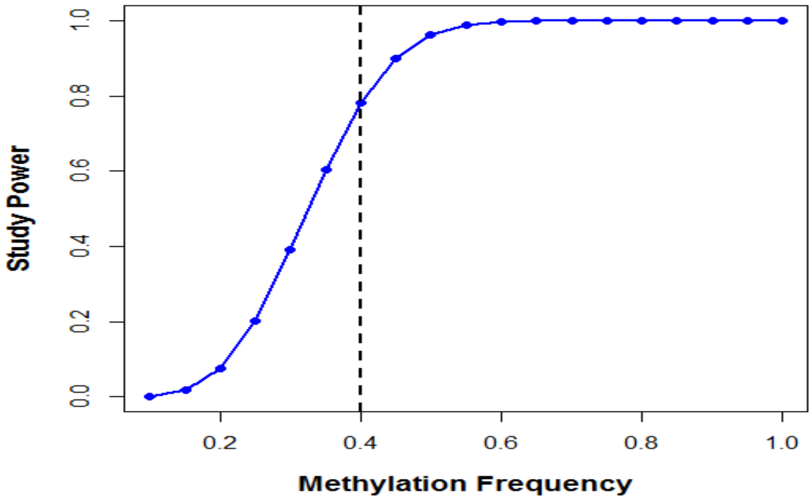

Supplementary Figure S2 Category of *de novo* methylated CpG sites in solid tumours

Liver cancer

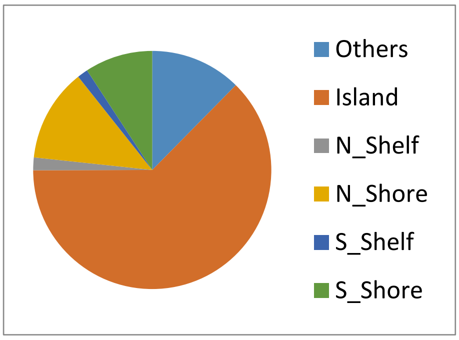

Rectal cancer

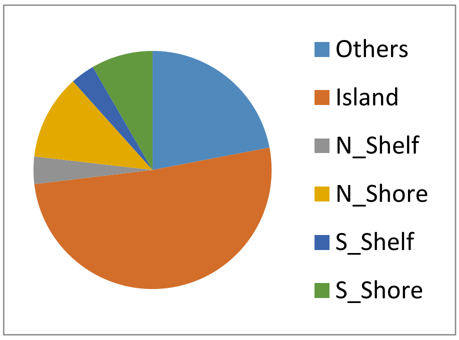

Colon cancer

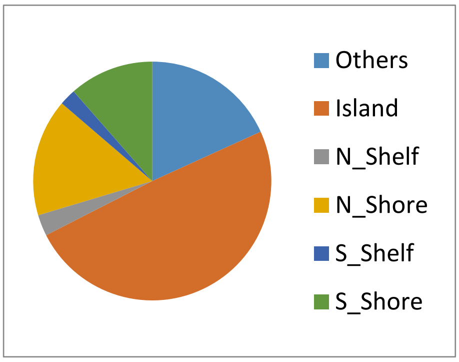

Lung squamous cell carcinoma

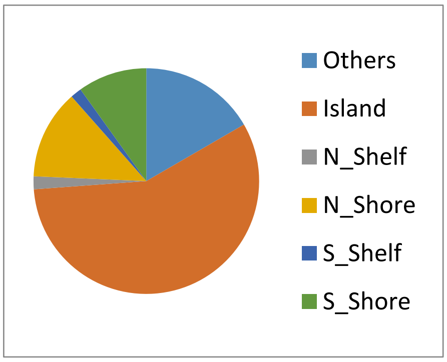

Thyroid cancer

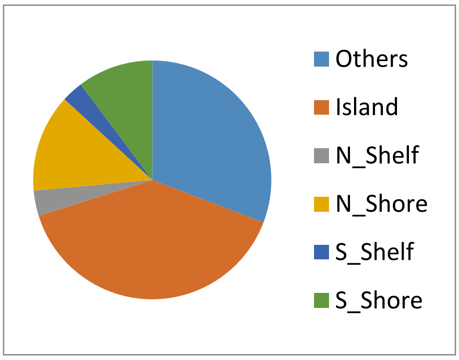

Kidney cancer

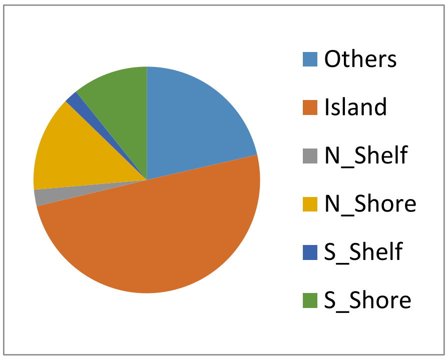

Invasive breast cancer

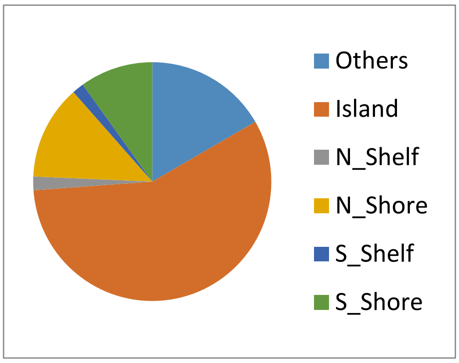

Lung Adenocarcinoma

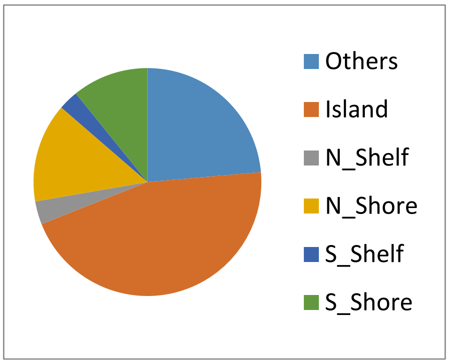

Pancreatic cancer

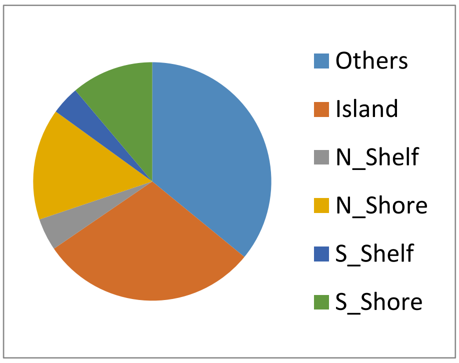

Prostate Cancer

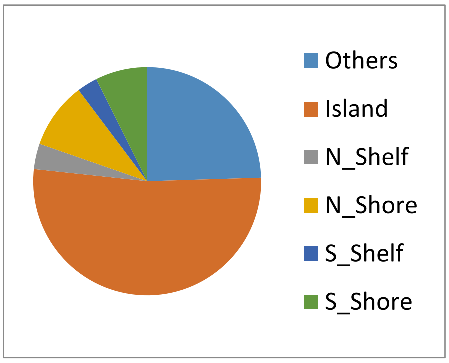

Head & neck cancer

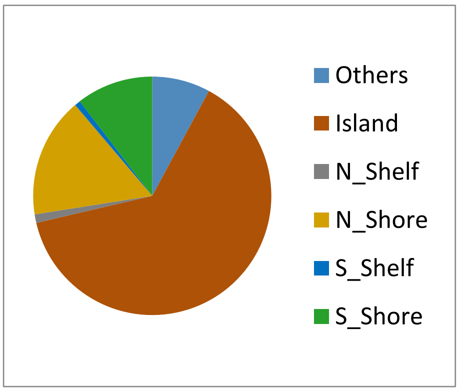

Supplementary Figure S3 Bisulphite pyrosequencing of six *de novo* methylated loci identified by Illumina Infinium Assay in NPC

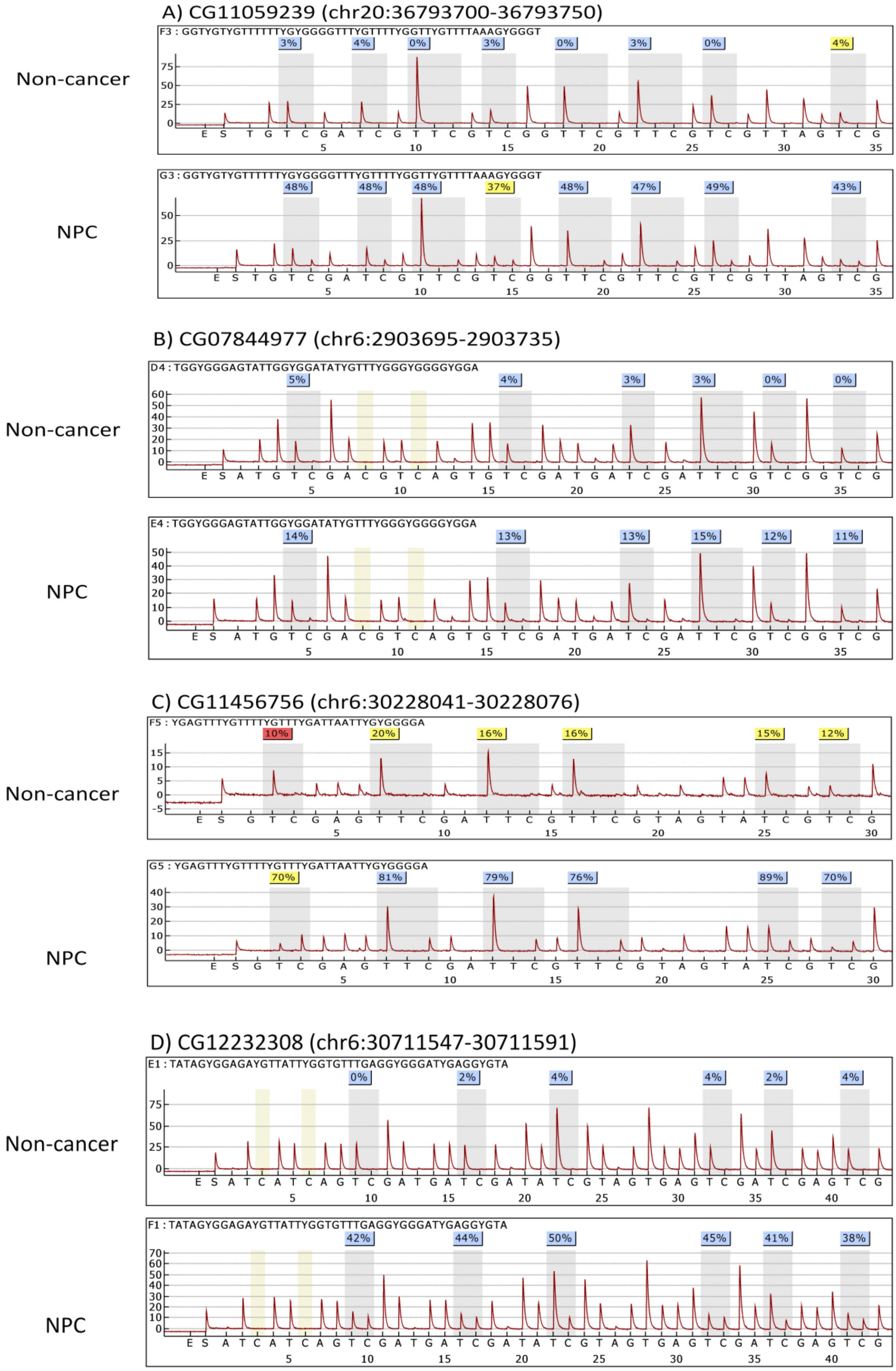

E) CG11664500 (chr6:133562458-133562497)

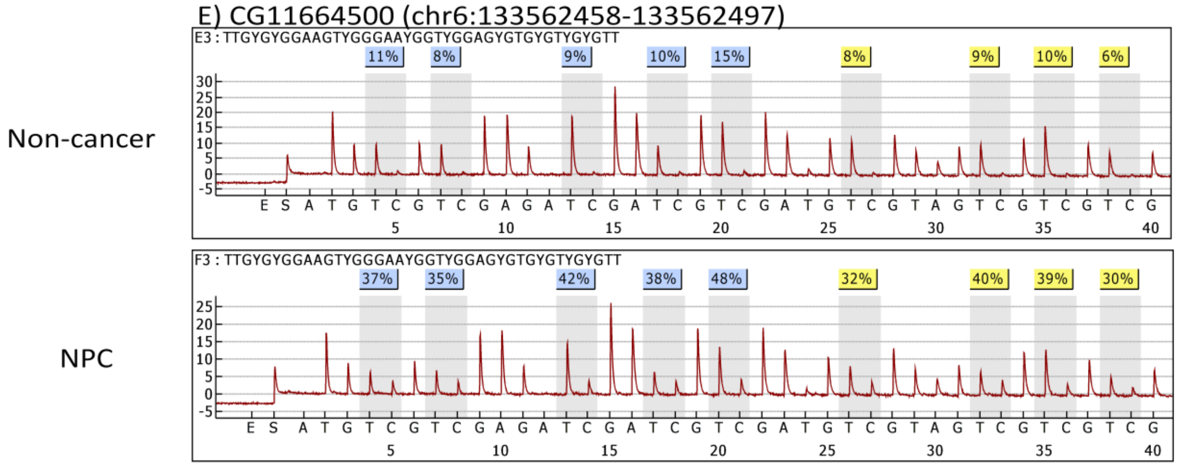

F) CG22903300 (chr6: 29760751-29760786)

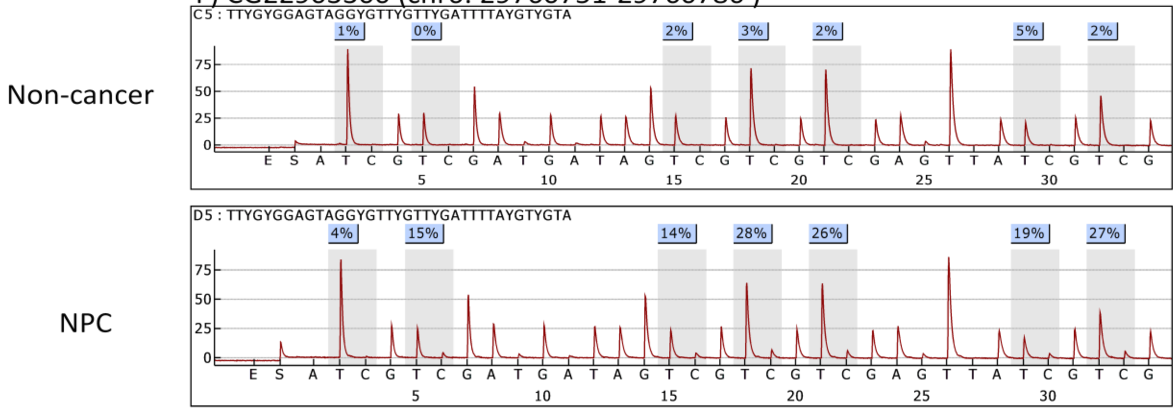

Supplementary Figure S4: Enrichment of H3K27me3 and H3K4me3 at the *de novo* methylated loci

Liver cancer

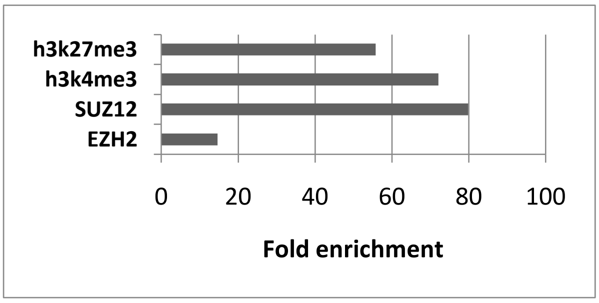

Rectal cancer

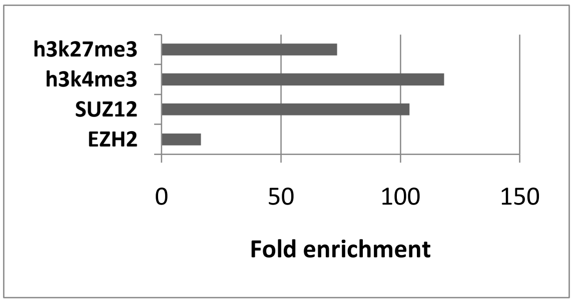

Colon cancer

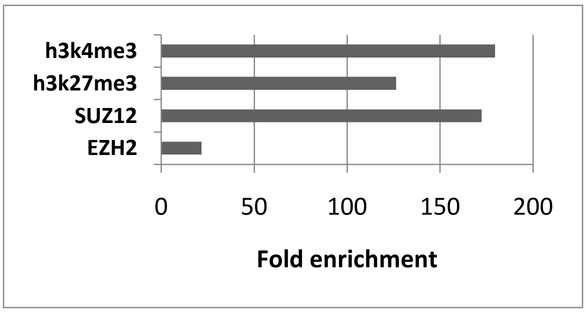

Lung squamous cell carcinoma

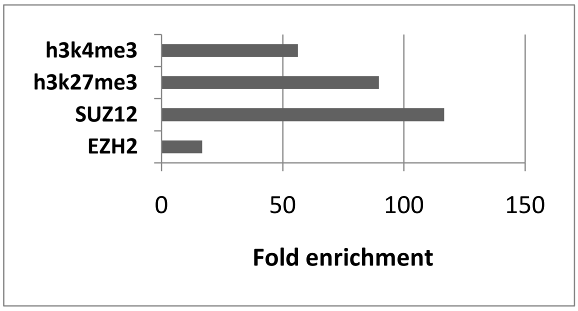

Thyroid cancer

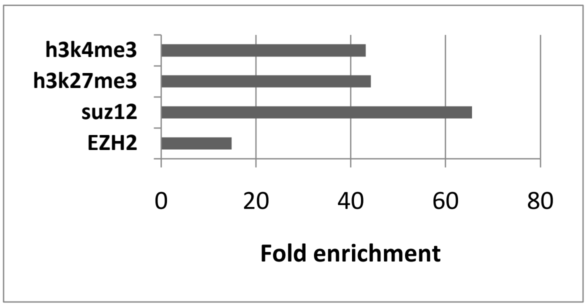

Kidney cancer

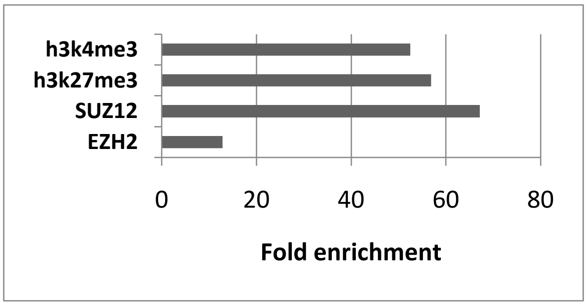

Invasive breast cancer

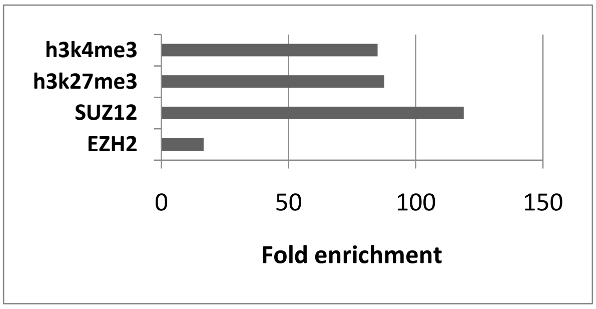

Lung adenocarcinoma

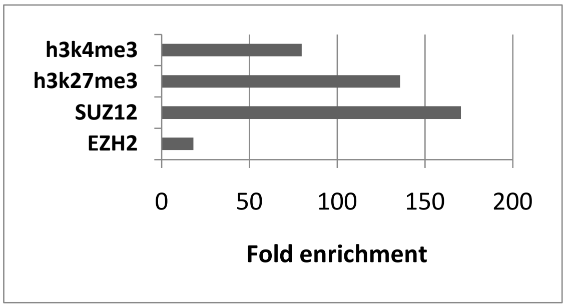

Pancreatic cancer

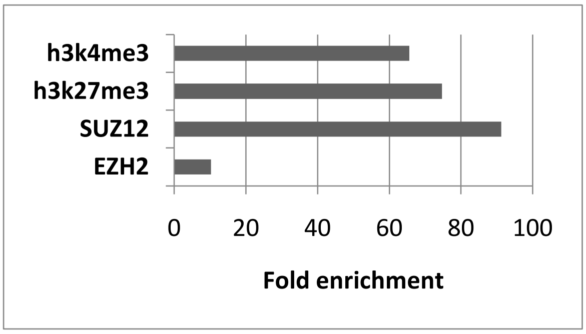

Prostate cancer

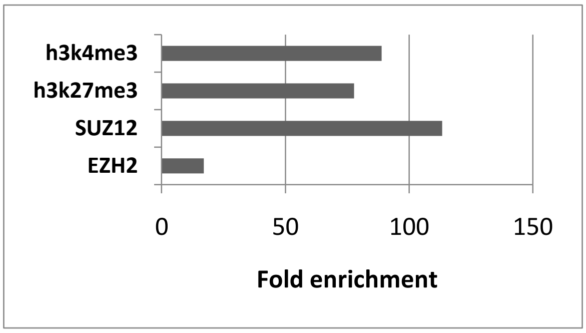

Head & neck cancer

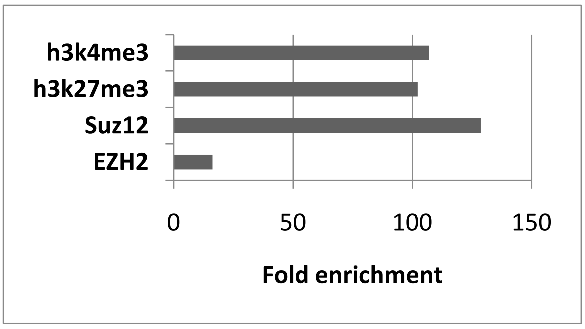

Supplementary Figure S5: Permutation test in the region-specific analysis in NPC

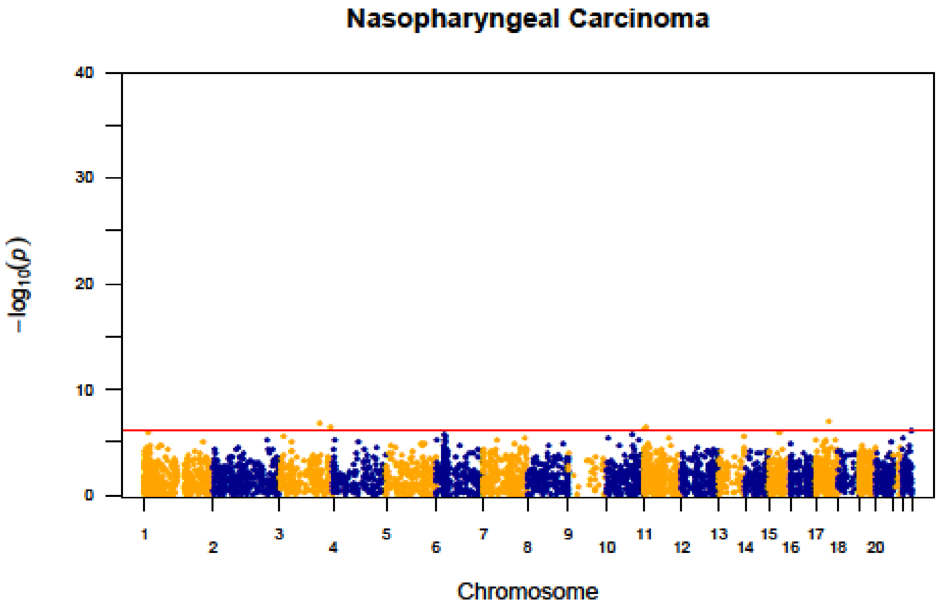

Supplementary Figure S6: Region-specific analysis of methylome data in

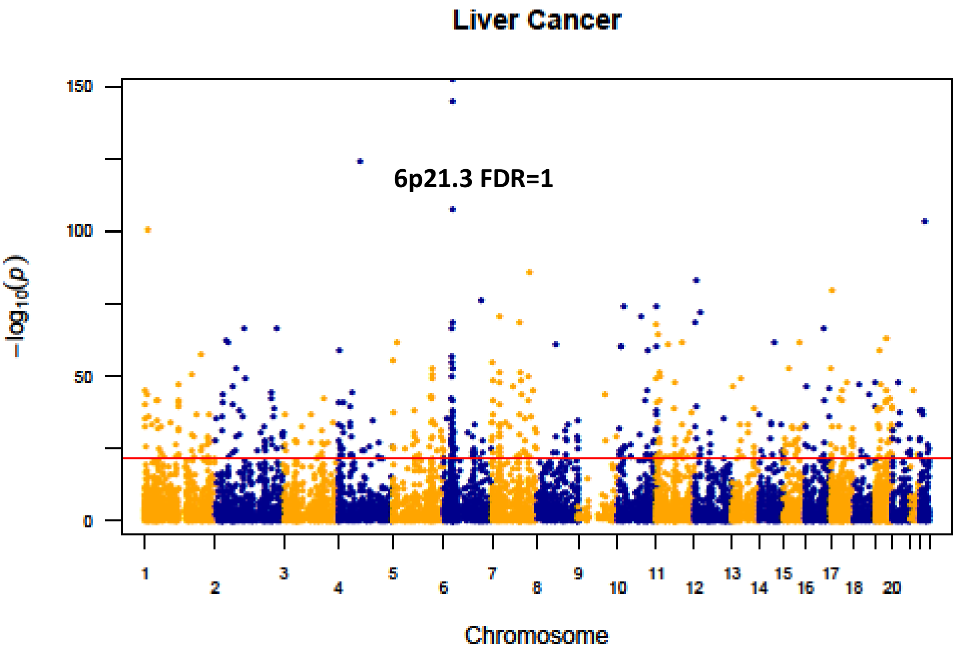

Rectal Cancer

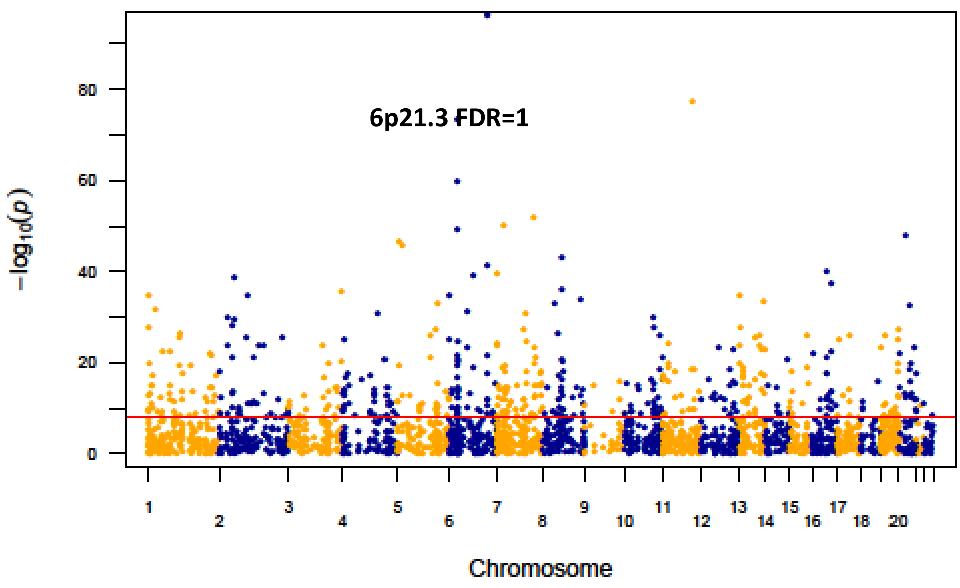

Lung squamous cell carcinoma

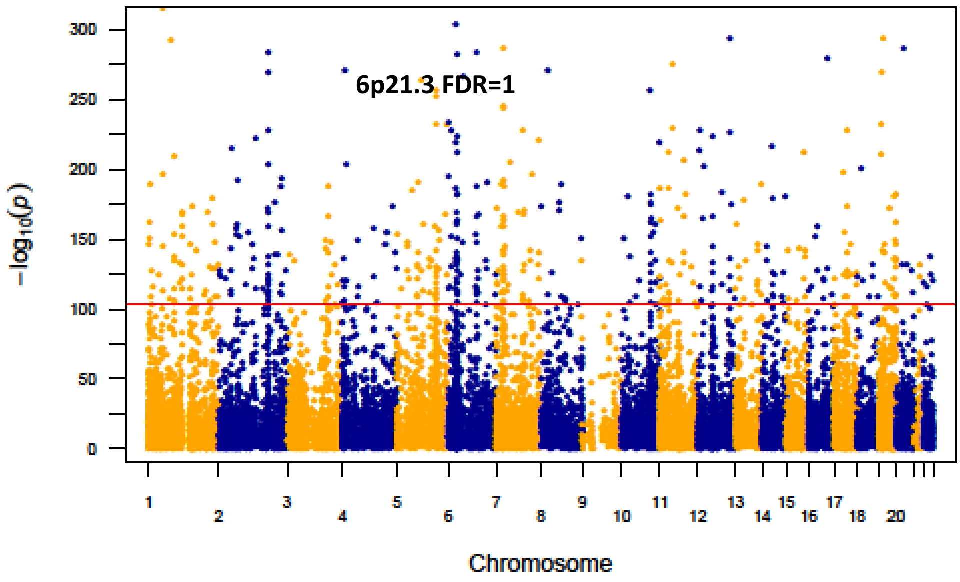

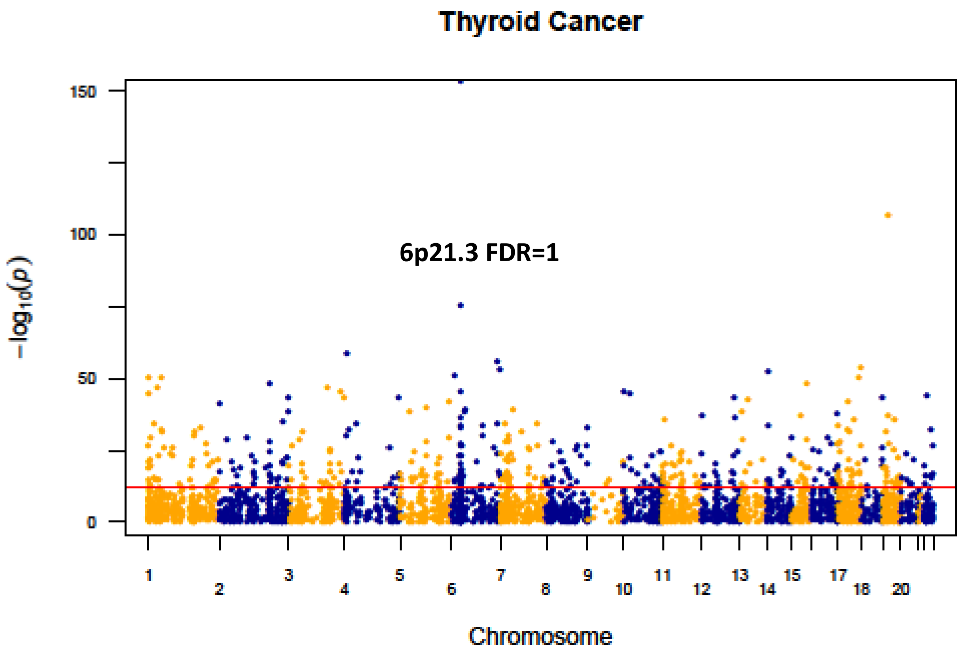
Thyroid Cancer
6p21.3 FDR=1
−log10(p)
Chromosome

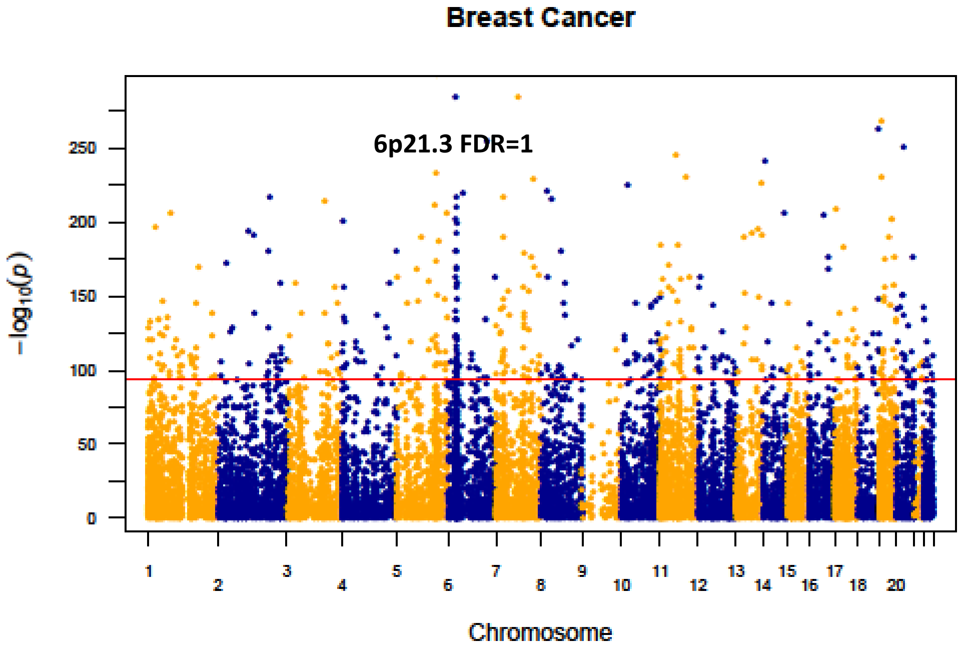
Breast Cancer
6p21.3 FDR=1
−log10(p)
Chromosome

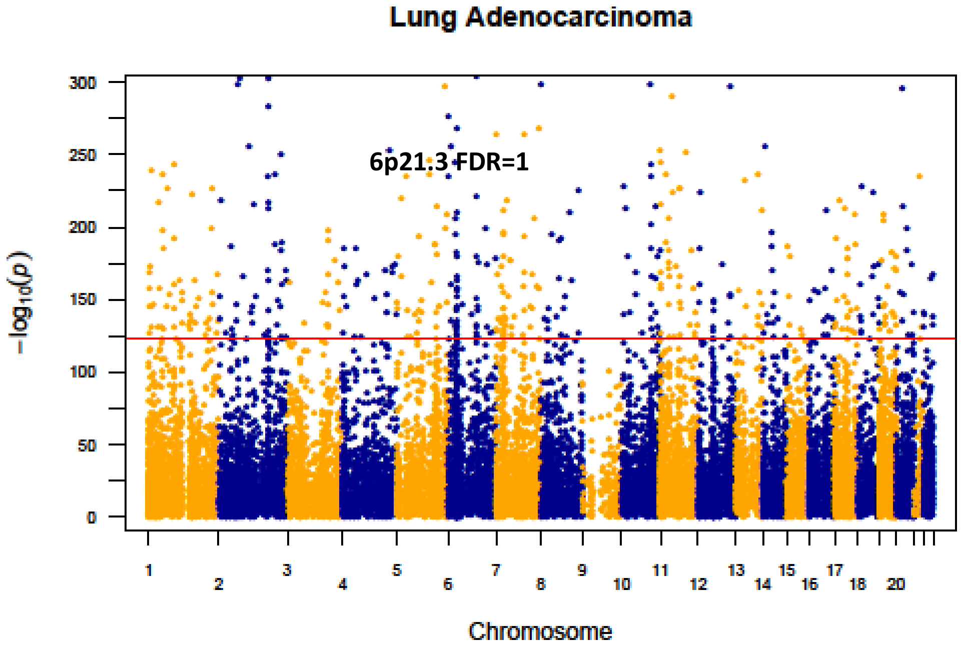
Lung Adenocarcinoma
6p21.3 FDR=1
$-\log_{10}(p)$
0
50
100
150
200
250
300
1
2
3
4
5
6
7
8
9
10
11
12
13
14
15
16
17
18
20
Chromosome

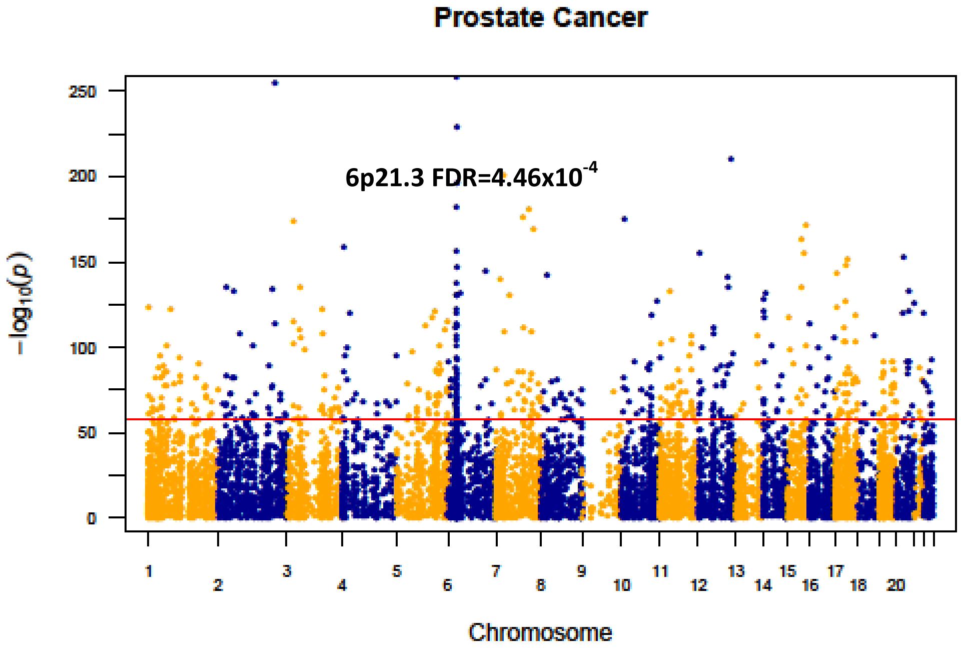
Prostate Cancer
6p21.3 FDR=4.46x10^-4
$-\log_{10}(p)$
0
50
100
150
200
250
1
2
3
4
5
6
7
8
9
10
11
12
13
14
15
16
17
18
20
Chromosome

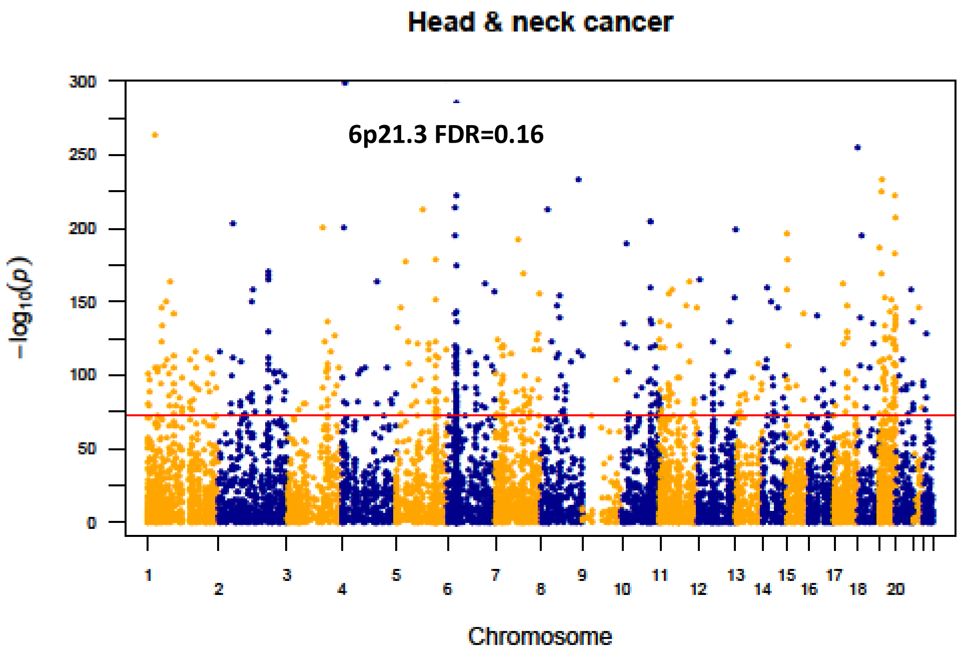

Supplementary Figure S7: Expression of B3GALT4 and disease-free survival in ovarian cancer (left) and lung cancer (right)*

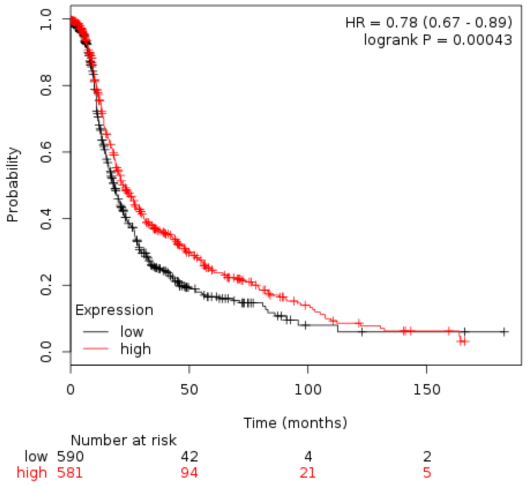

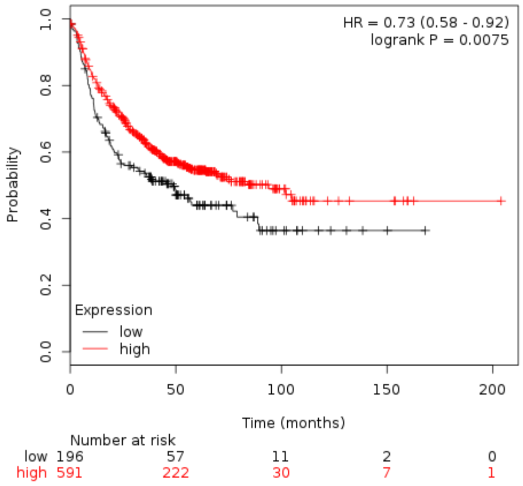

***The analysis is done by Kaplan Meier Plotter (http://kmplot.com/analysis/)**
